# Transcriptome-wide mRNA condensation precedes stress granule formation and excludes stress-induced transcripts

**DOI:** 10.1101/2024.04.15.589678

**Published:** 2024-05-02

**Authors:** Hendrik Glauninger, Jared A.M. Bard, Caitlin J. Wong Hickernell, Edo M. Airoldi, Weihan Li, Robert H. Singer, Sneha Paul, Jingyi Fei, Tobin R. Sosnick, Edward W. J. Wallace, D. Allan Drummond

**Affiliations:** 1 Graduate Program in Biophysical Sciences, The University of Chicago, Chicago, IL, USA.; 2 Interdisciplinary Scientist Training Program, The University of Chicago, Chicago, IL, USA.; 3 Department of Biology, Texas A&M University, College Station, TX, USA.; 4 Department of Biochemistry & Molecular Biology, The University of Chicago, Chicago, IL, USA.; 5 Fox School of Business and Management, Temple University, Philadelphia, PA, USA.; 6 Department of Anatomy and Structural Biology, Albert Einstein College of Medicine, Bronx, NY, USA.; 7 Gruss-Lipper Biophotonics Center, Albert Einstein College of Medicine, Bronx, NY, USA.; 8 Department of Neuroscience, Albert Einstein College of Medicine, Bronx, NY, USA.; 9 Institute for Biophysical Dynamics, University of Chicago, Chicago, IL, USA.; 10 Pritzker School of Molecular Engineering, University of Chicago, Chicago, IL, USA.; 11 School of Biological Sciences, University of Edinburgh, Edinburgh, Scotland, UK.; 12 Department of Medicine, Section of Genetic Medicine, The University of Chicago, Chicago, IL, USA.

## Abstract

Stress-induced condensation of mRNA and proteins into stress granules is conserved across eukaryotes, yet the function, formation mechanisms, and relation to well-studied conserved transcriptional responses remain largely unresolved. Stress-induced exposure of ribosome-free mRNA following translational shutoff is thought to cause condensation by allowing new multivalent RNA-dependent interactions, with RNA length and associated interaction capacity driving increased condensation. Here we show that, in striking contrast, virtually all mRNA species condense in response to multiple unrelated stresses in budding yeast, length plays a minor role, and instead, stress-induced transcripts are preferentially excluded from condensates, enabling their selective translation. Using both endogenous genes and reporter constructs, we show that translation initiation blockade, rather than resulting ribosome-free RNA, causes condensation. These translation initiation-inhibited condensates (TIICs) are biochemically detectable even when stress granules, defined as microscopically visible foci, are absent or blocked. TIICs occur in unstressed yeast cells, and, during stress, grow before the appearance of visible stress granules. Stress-induced transcripts are excluded from TIICs primarily due to the timing of their expression, rather than their sequence features. Together, our results reveal a simple system by which cells redirect translational activity to newly synthesized transcripts during stress, with broad implications for cellular regulation in changing conditions.

## Introduction

Cells must respond to changing environments to survive and thrive. When faced with a broad range of sudden maladaptive environmental changes—stresses—eukaryotic cells downregulate translation, induce stress-responsive transcriptional programs, and form cytosolic clusters of proteins and mRNA. When microscopically visible as foci colocalized with markers such as poly(A)-binding protein, these clusters are called stress granules (SGs)^1–7^,structures which are conserved across eukaryotes, yet still poorly understood. SGs are complex examples of biomolecular condensates, membraneless structures without defined stoichiometry which form by a range of processes and which concentrate specific types of biomolecules^8,9^. What precisely are stress granules composed of? How do they form and dissolve? What is their function, if any? What is the relationship between stress granule formation and the accompanying transcriptional and translational responses? All these questions remain active areas of inquiry.

Early work in multiple systems established that what are now recognized as stress granules recruit multiple RNA-binding proteins and translation initiation factors, along with pre-stress mRNA, yet exclude nascent mRNA produced during stress^10,11^. In mammalian cells, exclusion of two specific stress-induced heat shock protein mRNAs from SGs, HSP70 and HSP90^12,13^, along with nonspecific recruitment of untranslated mRNA^13^, matched prior work on heat shock granules in plants, which recruited mRNAs encoding housekeeping proteins but not those encoding newly synthesized heat shock proteins^6^.

Translation initiation serves as a focus of stress-dependent translational regulation and plays a central role in SG formation. Several translation initiation factors themselves are classic markers for stress granules, apparently as part of stalled translation initiation complexes preceding assembly of the large ribosomal subunit (60S) at the start codon. A wide range of stresses, from starvation to heat shock to oxidative stress, trigger phosphorylation of initiation factor eIF2α and subsequent repression of initiation for most mRNAs, and also cause SG formation. In certain cases, such as for heat shock in mammalian cells, preventing eIF2α phosphorylation is sufficient to prevent SG formation^1^. However, heat shock triggers SG formation by an eIF2α-phosphorylation-independent pathway in budding yeast^1,14^, indicating that eIF2a phosphorylation is not itself the trigger for SGs.

Instead, subsequent ribosome run-off, polysome disassembly, and the exposure of ribosome-free mRNA which serves as a template for SG assembly links initiation inhibition to SG formation^11,15^. Polysome disassembly has been called the “universal trigger” for SGs^16^. Consistent with the ribosome-free RNA template model, inhibitors of translation elongation which lock ribosomes on transcripts, such as cycloheximide (CHX) and emetine, inhibit SG formation, whereas an elongation inhibitor which causes ribosome release, puromycin, promotes SG formation^15,17^.

Recent work has provided extraordinary evidence, and a deeper biophysical foundation, consistent with a central role of ribosome-free RNA in stress granule formation. Transcriptome-scale study of the mRNA components of stress granules in both yeast and mammalian cells revealed that mRNA length is the overwhelming determinant of recruitment: long mRNAs accumulate in SGs, short mRNAs are excluded^4,18–20^. Long RNAs provide opportunities for multiple interactions necessary to form condensates—and thus for the multivalent interactions needed to drive biomolecular condensation, separation of a mixed solution of biomolecules into concentrated and dilute regions, now recognized as principle of cellular organization without membranes^8^. Increasing RNA length promotes RNA/protein phase separation in vitro by the stress-granule hub protein G3BP1^21,22^, and single-molecule studies show that mRNA length correlates with the dwell time of mRNAs on stress granules and other condensed structures^23^.

Yet these transcriptome-scale findings are in conflict with early results showing selective exclusion of stress-induced mRNAs from stress granules, a phenomenon not reported and, we show, not present in recent studies. Beyond length, only pre-stress translation levels or related features like codon bias have been identified as major correlates of recruitment^4^. Even this result is puzzling, given that SGs recruit nontranslating mRNAs after stress, not before stress, and yet no relationship between post-stress translation and SG recruitment has been reported to our knowledge. Meanwhile, stress-induced messages are translationally privileged during stress^24^, such that their recruitment to SGs—complex biomolecular condensates which concentrate nontranslating mRNA, among other defining features—would be paradoxical.

Finally, stress granules themselves have an unusual status as a biological phenomenon. With no associated function or phenotype for their specific disruption, they are presently defined solely by visual criteria, the presence of microscopically visible foci colocalizing with specific markers such as poly(A)-binding protein (Pab1 in budding yeast). Absence of foci is routinely interpreted as absence of stress granules. Yet biomolecular condensation of multiple RNA-binding SG components in vivo and in vitro in response to physiological stress conditions has been demonstrated^7,25–28^, and blocking SG formation with cycloheximide does not block in vivo condensation of Pab1^25^. Mild stresses trigger condensation without SG formation^25,29^. These results collectively indicate that stress-induced protein condensation is a distinct phenomenon from SG formation. They support a model in which stages of condensation occur prior to, and whether or not, stress granules eventually appear^30^. Whether RNA undergoes similar pre-SG stages remains unknown.

Here, using biochemical fractionation by sedimentation and RNA sequencing (Sed-seq), we show that virtually all pre-stress transcripts condense during stress regardless of their lengths, even in the absence of visible stress granules. At the transcriptome scale, stress-induced transcripts escape condensation and are robustly translated, confirming early anecdotal reports and contrasting with recent high-throughput results. Although some mRNAs which condense in response to one stress escape condensation in another, a surprisingly simple explanation rationalizes the differences: pre-existing transcripts condense, and newly produced transcripts escape condensation, permitting their preferential translation. We discover that specific endogenous transcripts are condensed prior to stress, only to be released from condensates upon stress for translational activation; indeed, condensation is pervasive in unstressed cells and results when translation initiation is inefficient. These translation-initiation-inhibited condensates (TIICs) are distinct from stress granules and potentiate SG formation. Together, these results show that mRNA condensation occurs even basally outside of stress and is measurable before visible stress granules form, expanding the importance of understanding mRNA condensation for cellular physiology in and outside of stress.

## Results

### Sed-seq enables measurement of transcriptome-scale mRNA condensation

**Figure 1:**
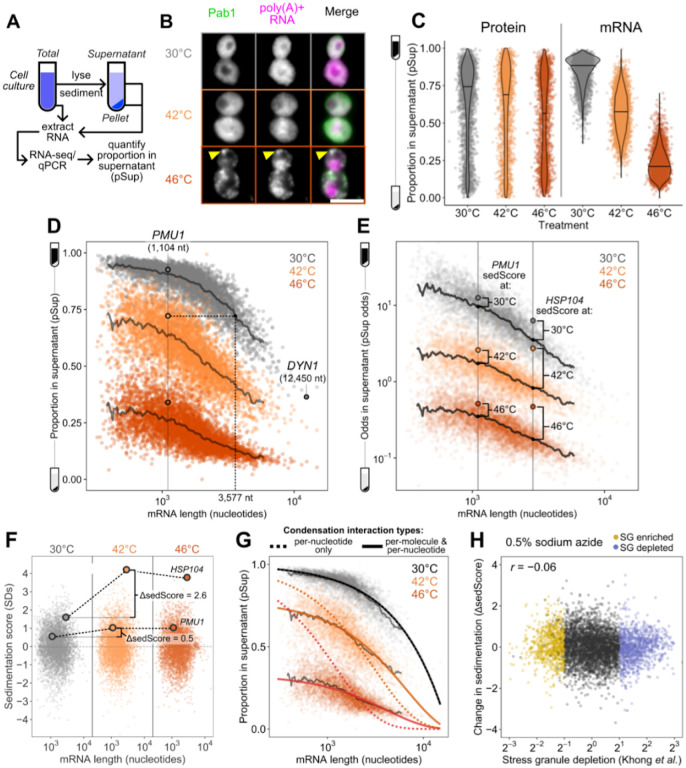
Most transcripts condense during stress, even in the absence of stress granules. (A) Analysis of mRNA condensation by sedimentation and RNA sequencing (Sed-seq) enables calculation of mRNA proportion in the supernatant (pSup) across conditions. (B) 15 minutes of heat shock induces stress granule formation at 46°C but not at 42°C, as marked by poly(A)-binding protein (Pab1-HaloTag) and FISH against poly(A)+ RNA (scale bar = 5μm). (C) Unlike protein condensation, which affects only a small proportion of proteins (data from Wallace *et al. Cell* 2015), virtually all transcripts condense during stress, even when stress granules are not apparent. (D) Transcript pSup decreases with length in unstressed cells due to sedimentation of large monomeric mRNPs. pSup globally decreases with 10 minutes of heat shock, reflecting stress-induced condensation. (E) Sed-seq data allow quantification of key features: absolute sedimentation pSup, and relative sedimentation (sedScore), which controls for effects of long transcript sedimentation. sedScore is calculated on a log-odds scale to enable comparisons between low- and high-pSup transcripts. (F) Transcripts show substantial and different changes in relative condensation during stress. *HSP104* mRNA increases 2.8 standard deviations in relative solubility (ΔsedScore = 2.6) while *PMU1* mRNA changes little (ΔsedScore = 0.5). (G) A simple clustering model (see Supplementary Text) captures average pSup and stress-induced changes. Clustering must involve interactions independent of RNA length (e.g. between 5’ or 3’ ends) to fit the data. (H) Condensation measured by Sed-seq (ΔpSup, lower = more condensation) correlates negatively with previously reported stress granule depletion (lower = more condensation) (Khong *et al. Mol Cell* 2017).

We previously used biochemical fractionation via sedimentation to isolate stress-induced protein condensates during heat shock in budding yeast^31,32^. To measure condensation of RNA, we coupled this sedimentation assay with RNA sequencing (Sed-seq) (Figure 1A). We collected and quantified transcript abundances in total, supernatant, and pellet fractions, and estimated the proportion of each gene’s transcripts in the supernatant (pSup) using a Bayesian mixture model^31^ validated by qPCR (Figure S1A). We included the chelating agent EDTA to disassemble polysomes which would otherwise sediment along with condensed mRNAs^25,33,34^. Sed-seq does not by design enrich for mRNA association with a particular type of RNA granule, such as stress granules or processing bodies, enabling an unbiased measurement of stress-induced RNA condensation. Here we use the broad term condensation to describe molecules interacting to form denser structures, without any presumption of the precise nature or mechanism of formation of these structures.

We first used Sed-seq to examine mRNA condensation transcriptome-wide in unstressed conditions (30°C) and after short heat shocks at 42°C and 46°C; as expected, 46°C produced clear stress granules, visible as poly(A)+ RNA colocalized with foci of poly(A)-binding protein (Pab1), while the milder 42°C shock did not produce stress granules (Figure 1 B). Sed-seq revealed large decreases in pSup across the transcriptome during heat shock, correlated with the intensity of the stress. Unlike stress-triggered protein condensation, which affects only a minority of the proteome^31^, virtually all transcripts show substantial condensation after stress (Figure 1C). Similar to protein condensation^14,31,35^, mRNA condensation occurs at 42°C even when SGs are not apparent.

Transcript length has been previously identified as the dominant determinant of mRNA recruitment to stress granules^36–38^. Such an effect seems intuitive because the likelihood of RNA-mediated molecular interactions naturally scales with length, consistent with the general importance of multivalency in biomolecular condensation^8^. In our data, long transcripts showed stronger sedimentation in all conditions, including when RNA was isolated from unstressed cells and when added, in purified form, to lysate (Figure S1B). We therefore sought to understand the origin of length-dependent sedimentation and its influence on downstream conclusions about the role of mRNA length and RNA-mediated multivalency in stress-triggered condensation. An extended treatment of our findings is provided in Supplementary Information, and we here focus on key insights, which differ markedly from conclusions of previous high-throughput studies.

Long transcripts—actually messenger ribonucleoprotein particles, or mRNPs—sediment when isolated from unstressed cells due to their mass (see Supplementary Text), without any need to invoke condensation (Figure 1D). Consequently, a transcript’s pSup is directly related to transcript length, whether or not that mRNA actually condenses. By spiking purified total mRNA from Schizosaccharomyces pombe into lysate from unstressed yeast cells, we verified that this length-dependence is recapitulated, and that this free mRNA largely remains soluble even when added to lysate from stressed cells in which most mRNA appears condensed (Figure S1B). The systematic relationship between pSup and mRNA length allows estimation of the size of stress-induced condensates in terms of the size of unstressed mRNPs with the same sedimentation behavior. For example, 1.1-kilobase *PMU1* transcripts sediment after 42°C heat shock as if they were more than three times their size. After 46°C shock, they sediment as if more than ten times their unstressed size, with pSup lower than the heaviest detected mRNP in unstressed yeast, the 12.4-kilobase transcript encoding dynein (*DYN1)* (Figure 1D). These apparent several-fold changes in size are lower bounds (see Supplementary Text) and their magnitude justifies the provisional interpretation of sedimentation changes as condensation.

Several quantitative features can be extracted from Sed-seq data. We start by plotting pSup in log-odds space, *log* (*Sup/Pellet*), to prevent compression at very high or low pSup values (Figure 1E). We then calculate the relative pSup compared to the mean for similar-length transcripts, quantified as a Z score (sedScore) (Figure S1C). The sedScore measures differences in mRNP mass and potential condensation within conditions and removes the effect of length-based sedimentation (Figure 1F). Finally, we calculate the change in sedScore after stress (ΔsedScore), which reports on stress-induced changes in condensation (Figure 1F). We noted that certain transcripts showed significant changes in response to stress, such as the molecular-chaperone-encoding *HSP104* mRNAs, which increase in relative solubility by more than 2.5 standard deviations upon 42°C shock (ΔsedScore = 2.8) while *PMU1* mRNAs increase by an insignificant 0.5 standard deviations (ΔsedScore = 0.5) (Figure 1F).

What interactions mediate condensation? A simple physics-derived model explains both the underlying length-dependence of pSup and the average increase in condensation across stresses (Figure 1G, Supplementary Text, Figure S1D,E). Two parameters govern condensation: the rate of interaction per nucleotide, and the rate of interaction per transcript. Per-nucleotide interactions model length-dependent interactions previously proposed to drive stress-granule recruitment, such as RNA-RNA interactions or interactions linked to RNA-binding proteins^19,36,37,39^; per-transcript interactions model length-independent interactions, such as those involving the 5’ cap or 3’ end. This model fits sedimentation transcriptome-wide (Figure 1G, solid lines), estimating both per-nucleotide and per-transcript parameters as non-zero (p < 2 × 10^−16).^ Importantly, length-independent interactions dominate the behavior of shorter mRNAs. Fitting average sedimentation with only per-nucleotide interactions dramatically underestimates the observed condensation of shorter mRNAs (Figure 1G, dotted lines). The median gene has transcript length 1,529 nt and more abundant mRNAs are on average shorter. We conclude that stress-triggered condensation is inconsistent with interactions solely mediated by RNA-RNA interactions.

How does stress-induced condensation compare to previous reports of the stress-granule transcriptome^36^? We initially compared ΔsedScore during heat stress to the reported stress-granule depletion based on pulldown and sequencing, and found that these measurements were uncorrelated (*r* = 0.01, Figure S1F). Because the previous study in yeast was done after 0.5% sodium azide treatment to induce stress granules, rather than heat shock, we treated cells with 0.5% azide and repeated Sed-seq. We found that the two measurements were slightly anticorrelated (*r* = −0.06, P<10^−5)^ (Figure 1H, Figure S1F). Because the previous study did not perform a non-stress control, we hypothesized that the inability to correct for length-based sedimentation created an artifactual enrichment for long transcripts. In support of this possibility, our Sed-seq results from unstressed cells reproduce the previously reported stress granule transcriptome to a high degree of accuracy (*r* = 0.8, Figure S1G). Whatever the reasons, Sed-seq produces results in sharp disagreement with previous work. We therefore asked whether meaningful biology might become apparent in these new data.

### Stress-induced mRNAs escape condensation and are preferentially translated

**Figure 2:**
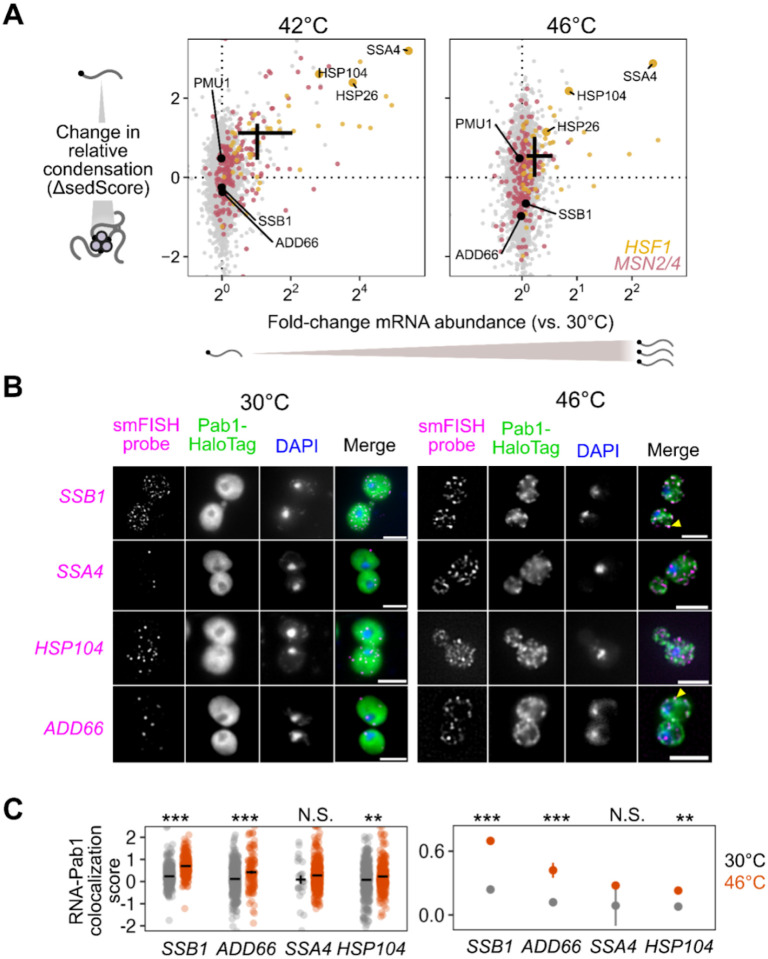
Induced transcripts escape condensation during heat shock. (A) Comparison of mRNA abundance changes during heat shock reveals that transcript induction quantitatively predicts escape from condensation, regardless of the primary transcription factor driving induction. (B) smFISH of induced (*SSA4/HSP104*) and uninduced *(SSB1/ADD66)* transcripts confirms that induced mRNA are not localized to Pab1-HaloTag marked stress granules. Scale bars are 5 μm. (C) Colocalization was quantified by comparing the intensity of the Pab1 channel in regions with mRNA foci to random regions in each cell. The colocalization score is plotted per cell (left) or as the mean of all cells (right). (Wilcoxon rank sum test, N.S.: *P* ≥ 0.05; **: *P* < 0.01; ***: *P* < 0.001)

The apparent escape of heat-shock-protein-encoding HSP104 transcripts from condensation during heat shock (Figure 1E,F) mirrors early reports of stress-induced transcript exclusion from stress granules^10–13^. With our transcriptome-scale data, we asked whether stress-induced transcripts were generally more likely to escape condensation. Indeed, genes regulated by the core heat shock response transcription factor Hsf1 strongly tend to escape condensation (ΔsedScore > 0) during heat shock (Figure 2 A, S2A,B, Wilcoxon rank sum test *P* < 10^−4)40^. Escape is not specific to Hsf1 targets, as most genes whose abundance is up-regulated by stress also escape condensation, including targets of Msn2/4, another stress-activated transcription factor (Figure 2A)^41^. We noted that the degree of induction correlated with the degree of escape, indicating that being regulated by stress-activated transcription factors was not the sole determinant of escape.

Stress-induced transcripts escape condensation even under conditions without apparent stress granules (e.g. 42°C). Are they also excluded from stress granules? To answer this question, we used single-molecule fluorescence in situ hybridization (smFISH)^42^ to examine the relative localization of transcripts to stress granules. We initially focused on two transcripts of nearly identical length, both encoding Hsp70 chaperones: *SSB1/2* transcripts, encoding a cytosolic Hsp70 species which is abundant in unstressed cells, and *SSA4*, encoding a stress-induced cytosolic Hsp70. We predicted that the induced *SSA4* transcripts would be excluded from stress granules. Consistent with our Sed-seq results, in 46°C heat-shocked cells, *SSB1/2* transcripts colocalized with stress granules marked by poly(A)-binding protein Pab1, while *SSA4* transcripts were largely excluded (Figure 2B). We then picked another pair of transcripts (*HSP104* and *ADD66*) to test the other observation from our Sed-seq data: that length was not a determining factor in stress granule association or exclusion. Indeed, induced long *HSP104* transcripts were excluded, and uninduced short *ADD66* transcripts colocalized (Figure 2 B). In order to quantify this observation, we calculated the intensity of the Pab1 channel in regions with mRNA and compared that to random regions around each cell (Methods). Reflecting the extent of the colocalization between the mRNAs and stress granules, *SSB1* and *ADD66* containing regions are strongly enriched for Pab1 signal upon stress, while *SSA4* and *HSP104* are only slightly enriched (Figure 2C). Together, Sed-seq and smFISH results form a consistent picture in which, regardless of length, stress-induced transcripts are excluded from condensates.

**Figure 3:**
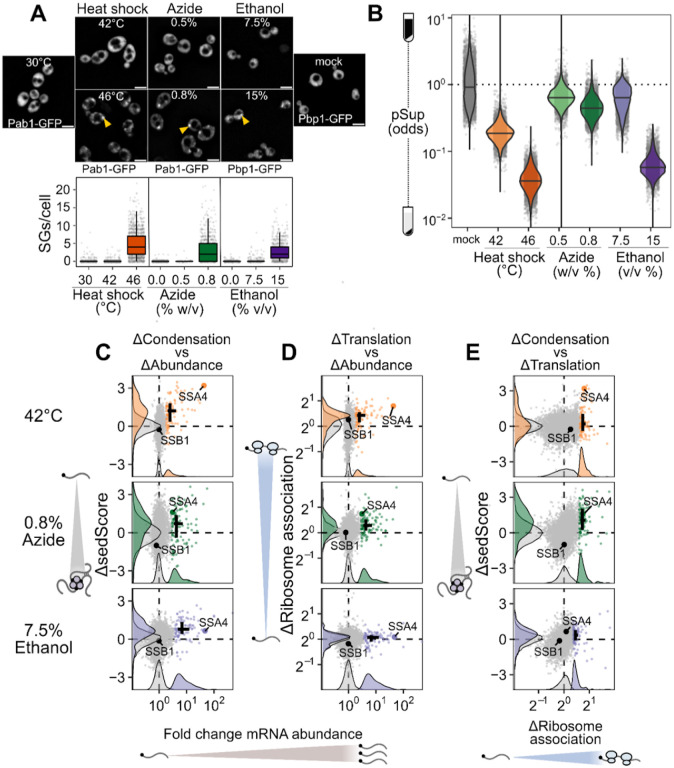
Newly transcribed and well-translated mRNAs escape condensation across stresses. (A) Severe, but not mild, stress induces visible SGs across multiple conditions. Scale bar is 5μm. (B) Both mild and severe stress induce transcriptome-wide sedimentation of mRNA, with the extent of pelleting correlating with the severity of the stress. (C) Across stresses, the most induced mRNA (top 100 induced transcripts are highlighted) escape from condensation. (D) Polysome-seq was used to measure the stress-induced change in ribosome association (top 100 induced transcripts are highlighted). After heat shock and azide stress, the most induced transcripts also have increased relative translation. (E) Directly comparing changes in translation and sedimentation (top 100 translationally upregulated transcripts are highlighted) shows that well-translated messages during stress tend to escape condensation.

Is the escape of induced transcripts from condensation specific to heat shock? To answer this question, we carried out Sed-seq on cells exposed to different stresses: treatment with sodium azide (NaN_3)_, a standard trigger for stress granules^36,43–45^, or with high concentrations of ethanol, a physiological condition for budding yeast which is also known to trigger granules^46^ (Figure 3A). Following previous literature, we tracked SG formation using Pab1-GFP for heat shock and NaN_3_ stress, and Pbp1-GFP for ethanol stress^31,43,46^. Across all three stresses, only severe stress triggered visible granule formation, while transcriptome-wide mRNA condensation was dose-dependent (Figure 3B). We find little evidence for increased stress-induced condensation of long transcripts for any of these stresses (Figure S3A).

Strikingly, stress-induced transcripts relatively escaped condensation across all three stresses (Figure 3C, S3C) as quantified by ΔsedScore. This result, now with transcript-specific precision, echoes early results showing exclusion of nascent transcripts from SGs^10,11^. In contrast, induced transcripts are not depleted from the previously reported SG transcriptome (Figure S3B)^36^. Do the same transcripts escape mRNA condensation in response to different stresses? Comparison of the ΔsedScore’s between stresses addresses this question. We compare the transcripts which are uniquely induced during heat shock, azide and ethanol stress, finding that a transcript generally escapes condensation if it is induced in that specific stress (Figure S3C). This is particularly apparent for the comparison between temperature and ethanol stresses.

To what extent does mRNA translation correlate with escape from condensation? Because our results so clearly match early observations of untranslated-mRNA condensation and nascent-transcript escape, we measured mRNA-ribosome association transcriptome-wide by isolating and sequencing mRNA from polysome gradients, quantifying the stress-induced change in ribosome association on each transcript (Polysome-seq)^47^. In each of the three stresses, induced transcripts tended to be preferentially translated (Figure 3D, S3E). Similarly, preferentially translated transcripts tend to escape condensation (Figure 3E, S3F). Transcriptional induction, escape from condensation, and increased translation co-vary in each stress condition, indicating a functional role for condensation in translational repression of pre-existing transcripts. However, these results do not reveal the direction of causality.

The observation that stress-induced transcripts escape condensation is consistent with a model in which newly produced transcripts are protected from condensation for some time during stress, regardless of their identity. This temporal escape model predicts transcript exclusion will correlate with the level of induction, which is directly related to the proportion of transcripts which are new during stress, assuming degradation can be neglected. A major alternative to the new-transcript model is that sequence-encoded mRNA features, such as structure or the presence of specific motifs or untranslated-region (UTR) binding sites, determine escape. This alternative model predicts that transcripts will escape condensation independent of induction level. Sed-seq data are consistent with the new-transcript model, showing escape from condensation strongly depends on induction level (Figure 3C, S3C). Even transcripts in the same regulons (Hsf1 and Msn2/4 during heat shock) show varying levels of escape dependent on their induction.

**Figure 4:**
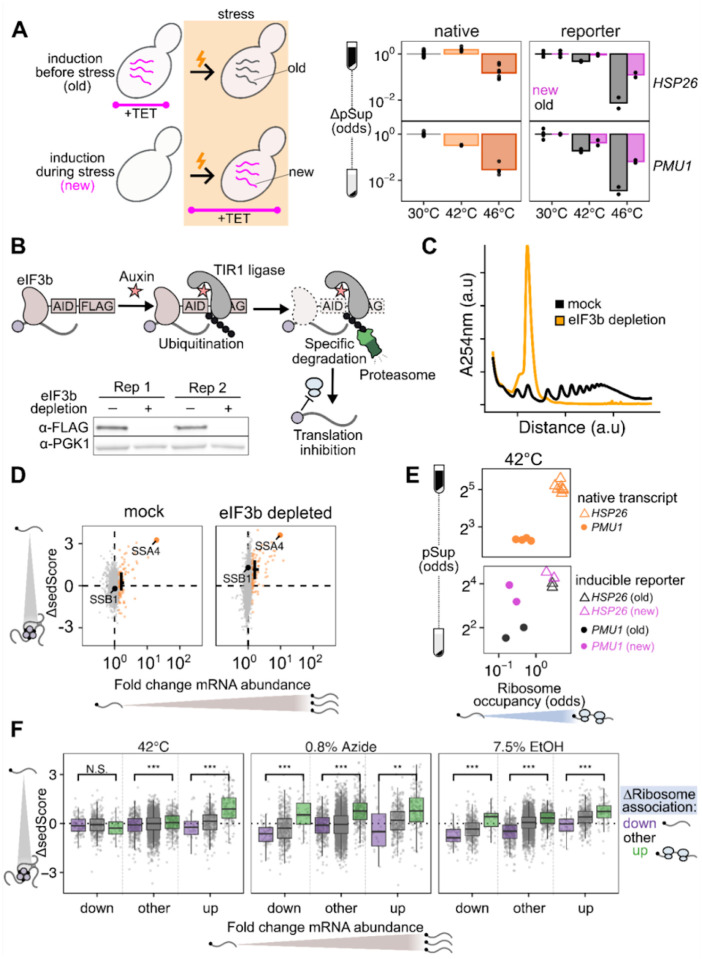
Translation and induction are independently sufficient to promote escape from condensation. (A) pSup was measured by qPCR for inducible reporter transcripts with sequences derived from an induced transcript *(HSP26)* or an uninduced transcript *(PMU1)*. Regardless of transcript sequence, “new” transcripts sediment less than “old” transcripts, showing that induction during stress is sufficient to drive escape from condensation. (B) The auxin-induced degradation system was used to deplete the translation initiation factor elF3b. (C) Depletion of elF3b lead to translational collapse as measured by polysome profiles. (D) Even in the absence of translation initiation, stress-induced transcripts still escape condensation after 10 minutes of 42°C stress (highlighted: top 100 induced transcripts per condition). (E) The *HSP26*-derived reporter transcript is better translated than the *PMU1* reporter, as measured by qPCR analysis of ribosome association using sucrose cushions. Despite its poor translation, the newly induced *PMU1* reporter still relatively escapes condensation. (F) Analysis of data in Figure 3 dividing transcripts into the top 10% most up or down regulated transcripts by abundance and translation state. Translation is well correlated with escape from condensation across induction levels and stresses. (Wilcoxon rank sum test, N.S.: *P* ≥ 0.05; **: *P* < 0.01; ***: *P*< 0.001)

If timing of transcript production largely drives escape from condensation, then it should be possible to construct and express synthetic transcripts whose condensation is determined only by when their expression occurs. We built inducible reporters with regulatory regions (5′ and 3′ UTRs) from genes which are heat-induced (*HSP26)* and heat-insensitive (*PMU1*, whose condensation behavior follows the bulk pre-stress transcriptome). We chemically induced each reporter before and during heat shock, and measured their condensation behavior via sedimentation with qPCR. Both reporters were uncondensed at 30°C, and condensed at 42°C and 46°C when expressed prior to heat shock. Both, however, showed substantially reduced condensation when newly expressed during heat shock (Figure 4A). These results provide further evidence that the timing of expression is a primary determinant of a transcript’s condensation fate. Transcripts which are newly produced during stress will escape condensation to a significant degree, independent of their sequence features. On the other hand, transcripts produced before stress, even if they contain the sequence of a stress-induced gene such as *HSP26*, will nevertheless condense during stress.

Given the clear relationship between transcript induction and escape from condensation, we sought to understand how translation fits into this model. First, we asked whether active translation is required for transcripts to escape condensation. We generated a strain of yeast with an auxin-inducible degron (AID) tag on the C-terminus of eIF3b, a subunit of the essential initiation factor eIF3^48,49^. Western blotting confirmed successful degradation (Figure 4B), which resulted in profound reduction in global translation, as evidenced by polysome collapse (Figure 4C). We then performed Sed-seq on samples heat shocked after two hours of mock treatment or depletion of eIF3b. Even in cells with translation initiation blocked by eIF3b depletion, newly transcribed messages escape condensation, as highlighted with the black cross indicating the mean ΔsedScore of induced transcripts (Figure 4D). We conclude that escape from condensation by newly transcribed mRNAs can occur independent of their translational status.

Transcripts do not require active translation to escape condensation, but does their translational state affect how much they condense? To address this, we revisited the TET-inducible reporter system described above and determined the translational state of the reporter transcripts. We measured ribosome occupancy by spinning lysate through a sucrose cushion and quantifying the ribosome-free abundance in the supernatant and the ribosome-bound abundance in the pellet, after correcting for condensed mRNA which pellets even in EDTA buffer (Figure S4A–D). We found that, after 20 minutes of 42°C stress, the *HSP26* reporter had high levels of ribosome occupancy while the *PMU1* reporter had low ribosome occupancy regardless of whether the transcripts were new or old (Figure 4E). This translational difference matched the behavior of the native transcripts; native *HSP26* transcripts have a higher ribosome occupancy than native *PMU1* transcripts across conditions. Correspondingly, for both old and new transcripts, the well-translated *HSP26* reporter had a higher pSup than the poorly translated *PMU1* reporter at 42°C. This result is reflected in the transcriptome-wide data: across stresses, transcripts with increased translation were more likely to escape condensation than those with repressed translation (Figure 4F). This held true for the top 10% most-induced and top 10% most-repressed transcripts in ethanol and azide stresses, confirming that the influence of translation on condensation is layered on top of the newness of a transcript during stress. Although active translation is not required for a transcript to escape condensation, more translation can lead to more escape. This finding now invites the question: what is the fate of transcripts that are blocked in translation?

### Translation inhibition-induced condensates (TIICs) of mRNAs precede stress granule formation and form in the absence of stress

**Figure 5:**
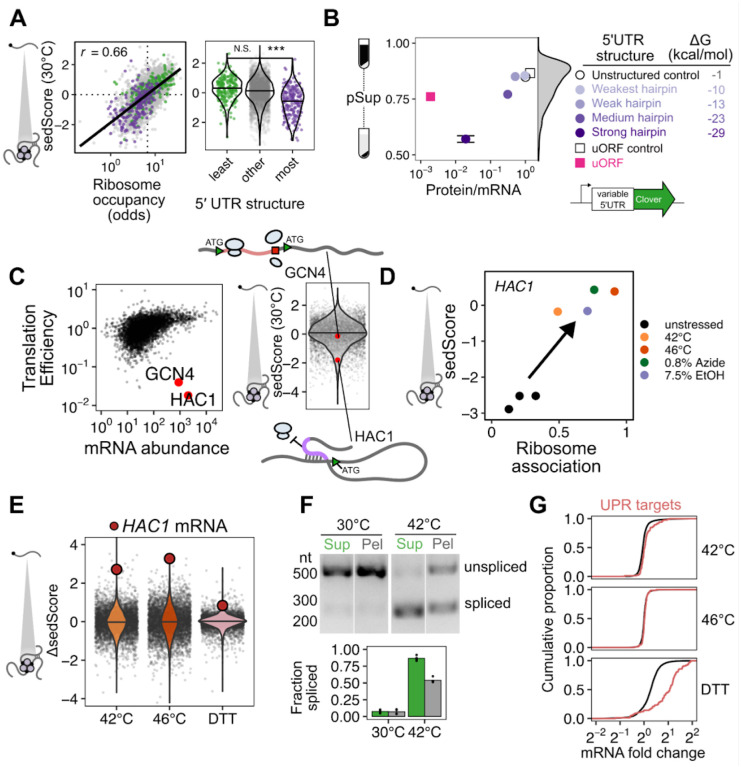
Translation-initiation-inhibited condensates (TIICs) form in the absence of stress. (A) Left: Ribosome occupancy (fraction of a transcript bound to at least one ribosome) in unstressed cells correlates well with length-normalized sedimentation (sedScore). Right: The amount of computationally predicted structure in the 5’ UTR of transcripts predicts their sedScore. (B) Sedimentation reporters with variable strength 5’ UTR hairpins or uORFs confirm the impact of translation initiation block on RNA condensation. Translation was quantified by the ratio of steady-state protein levels to mRNA abundance. Increased 5’ UTR structure or the presence of a uORF leads to decreased translation and increased sedimentation. (C) *HAC1* and *GCN4* transcripts are both abundant and poorly translated, using data for translational efficiency from Weinberg et al. 2016. In unstressed cells, *HAC1* mRNA has a low sedScore while *GCN4* mRNA exhibits nearly average sedimentation. (D) Across stresses, *HAC1* mRNA is translationally activated and also increases its relative sedimentation. (E) *HAC1* mRNA becomes less condensed during heat shock and DTT treatment. (F) 42°C treatment leads to splicing of *HAC1* mRNA as measured by RT-PCR, and the spliced form sediments less than the unspliced form. (G) UPR targets are slightly upregulated at 42°C and strongly upregulated in response to DTT treatment.

To this point, we had focused on stress-induced condensates. However, in examining the transcriptome-scale data we noted that at both 30°C and 42°C there was a striking correlation (*r* = 0.49 and 0.51, respectively, P < 10^−6)^ between sedScore and transcript abundance (Figure S5A). That is, even in the absence of stress, abundant transcripts sediment less than rare transcripts. What could account for this observation? One feature of abundant transcripts is that they tend to be well-translated (Figure S5B). Indeed, the sedScores of transcripts during basal growth (at 30°C) showed an even stronger correlation with their translation state, as measured by ribosome occupancy—the fraction of an mRNA bound to at least one ribosome (Figure 5A, Spearman *r* = 0.66, P < 10^−6)^. To further test this result, we divided transcripts by the strength of the secondary structure in their 5′ UTR, a feature known to predict the translation initiation efficiency of a transcript^50^ (Figure 5A). Transcripts with the least and most predicted structure in their 5′ UTR had, respectively, higher and lower sedScores than the bulk transcriptome. To explain this observation, we hypothesized that even during basal growth, poor translation initiation directly induces mRNA condensation which is observable in our sedimentation assay.

Accordingly, we asked whether we could recapitulate our in vivo observations of transcript-specific condensation using a series of synthetic mRNAs encoding the fluorescent protein Clover with progressively stronger translation initiation blocks created by hairpins in their 5′ UTR^51^. The hairpin series blocked translation initiation as measured by the ratio of fluorescence intensity to mRNA abundance, with more-stable hairpins more completely blocking translation (Figure 5B). As predicted, these constructs exhibited increased sedimentation which correlated with their translational efficiency (Figure 5B), demonstrating that in unstressed cells, a single species of translation initiation-inhibited mRNA forms sedimentable condensates.

Condensation of untranslated RNAs is consistent with a standard model for stress granule formation in which ribosome-free mRNA triggers condensation through RNA-mediated interactions^16,21,22,52^. Are poorly translated transcripts condensing in unstressed cells because they lack ribosomes, or for some other reason? To investigate this further, we turned to a pair of exemplary endogenous transcripts.

Among abundant transcripts in yeast—present in an estimated one copy or more per cell—two transcripts, *HAC1* and *GCN4*, stand out as being strongly translationally repressed in unstressed cells, either using translation efficiency data^50^ (Figure 5C) or ribosome occupancy data reported here (Figure S5B). *HAC1* encodes the master regulator of the unfolded protein response (UPR) while *GCN4* encodes the master regulator of the amino acid starvation response. *GCN4* has a length comparable to *HAC1* (1465 and 1197 nucleotides, respectively), and both are largely ribosome-free due to distinct mechanisms. Translation of upstream ORFs on the *GCN4* mRNA results in translation initiation but without translation of the main coding region^53^, while RNA-RNA interactions within the *HAC1* mRNA block translation initiation^54^.

However, the solubility of *GCN4* is typical for its length, whereas the solubility of *HAC1* is significantly lower than the mean of abundant transcripts (Figure 5C, S5C). *HAC1* transcripts sediment as if they were nearly four times the size of similar-length abundant mRNPs (Figure S5D), strongly hinting that condensation of multiple mRNPs, rather than merely additional mRNP mass, drives their sedimentation.

Consequently, ribosome-free mRNA, previously called the “universal trigger” of stress granule formation^16^, cannot explain the sedimentation differences between *GCN4* and *HAC1*. Both species of transcripts are largely ribosome-free, yet only *HAC1* condenses. We replicated this difference with synthetic reporters that differed in their mechanisms of translation repression. A synthetic uORF construct built from the *GCN4* 5′ UTR yielded substantially less condensation than the most stable hairpin construct, despite showing far stronger translational repression (Figure 5B). A control construct with five point mutations disrupting the start codon in each uORF^55^ promoted translation of the main open reading frame, as expected, and only modestly increased transcript solubility.

Together, these results form a coherent picture: a blockade in translation initiation, rather than the consequent exposure of ribosome-free mRNA, causes condensation affecting virtually the entire transcriptome under non-stress conditions. Because these non-stress condensates do not form microscopically visible foci and occur in the absence of stress, and thus are not stress granules, we refer to them as *translation-initiation-inhibited condensates*: TIICs (“ticks”).

### TIIC dissolution corresponds to translation initiation for UPR regulator HAC1

We noticed that *HAC1* mRNA, among the least-soluble transcripts in unstressed cells at 30°C (sedScore = −2.89), jumped by roughly three standard deviations in relative solubility upon a 10-minute heat shock at 42°C (ΔsedScore = 2.71) or 46°C (ΔsedScore = 3.27). The translation initiation inhibition of *HAC1* is relieved by mRNA splicing in the cytoplasm, leading to translation of the encoded Hac1 transcription factor, its nuclear import, and subsequent UPR activation. This process was originally reported to be insensitive to heat stress using a 37°C shock^54^. Recently, a minor induction of *HAC1* splicing has been observed after hours of growth at 39°C^56^. The phenomena we observe above 42°C led us to hypothesize that this more robust heat shock caused dissolution of TIICs containing *HAC1* mRNA corresponding to relief of translation initiation inhibition by splicing. Indeed, across stresses, *HAC1* is both better translated and less condensed (Figure 5D). Multiple predictions follow: 1) *HAC1* TIIC dissolution should occur during activation by other UPR triggers; 2) *HAC1* should be spliced in response to the short heat shocks which trigger TIIC dissolution; 3) if *HAC1* mRNA is translated, the resulting Hac1 transcription factor should drive transcription of UPR genes.

We tested each of these predictions in turn. First, we performed Sed-seq on cells treated with DTT, a standard UPR trigger. Confirming our prediction, *HAC1* mRNA showed among the strongest changes in relative solubility across the entire transcriptome upon DTT treatment (Figure 5E).

Second, we examined *HAC1* splicing in response to a 8-minute, 42°C heat shock. Before shock, *HAC1* mRNA was unspliced, running as a single large band. After shock, the spliced form of *HAC1* appeared as a smaller band (Figure 5F), confirming our second prediction. Under these conditions, *HAC1* is not completely spliced, which allowed us to make another crucial observation: the spliced form of *HAC1* partitioned disproportionately into the soluble fraction relative to the unspliced form (Figure 5F), again consistent with *HAC1*’s formation of TIICs before stress and stress-induced dissolution.

Third, we looked for transcription of UPR genes at 42°C, as identified in Kimata et al 2006^57^. We observed a slight but unmistakable induction after a 10-minute 42°C shock (Figure 5G, Wilcoxon rank sum test P < 10^−6)^ Based on this positive result, we predicted that other heat-shock data would show induction of the UPR at 42°C. Indeed, data from a systematic study of the heat shock response in budding yeast^58^ revealed that UPR targets were not induced by a 37°C shock for 10 or 30 minutes (Wilcoxon test P values 0.15 and 0.70, respectively) as previously reported^54^, but were significantly induced by 42°C shocks of 10 or 30 minutes (Wilcoxon test P values < 10^−3^ in both cases) (Figure S5E).

Together, these results support a simple and previously unappreciated sequence of events during *HAC1* activation: *HAC1* mRNA, in TIICs before shock, decondenses and is spliced, permitting translation of the Hac1 transcription factor protein which then drives UPR regulon transcription. Both DTT and short-term heat shock at 42°C produce this behavior.

Dissolution of *HAC1* TIICs and subsequent translation initiation at 42°C occurs while most other pre-stress transcripts experience the opposite effects, a blockade in translation initiation and formation of TIICs. The TIIC model predicts that globally blocking translation initiation, even in the absence of heat shock, should trigger transcriptome-wide mRNA condensation distinct from stress-granule formation. We therefore set out to test this prediction.

### Blocking translation initiation at distinct steps causes mRNA condensation and implicates an upstream, competitive step

**Figure 6:**
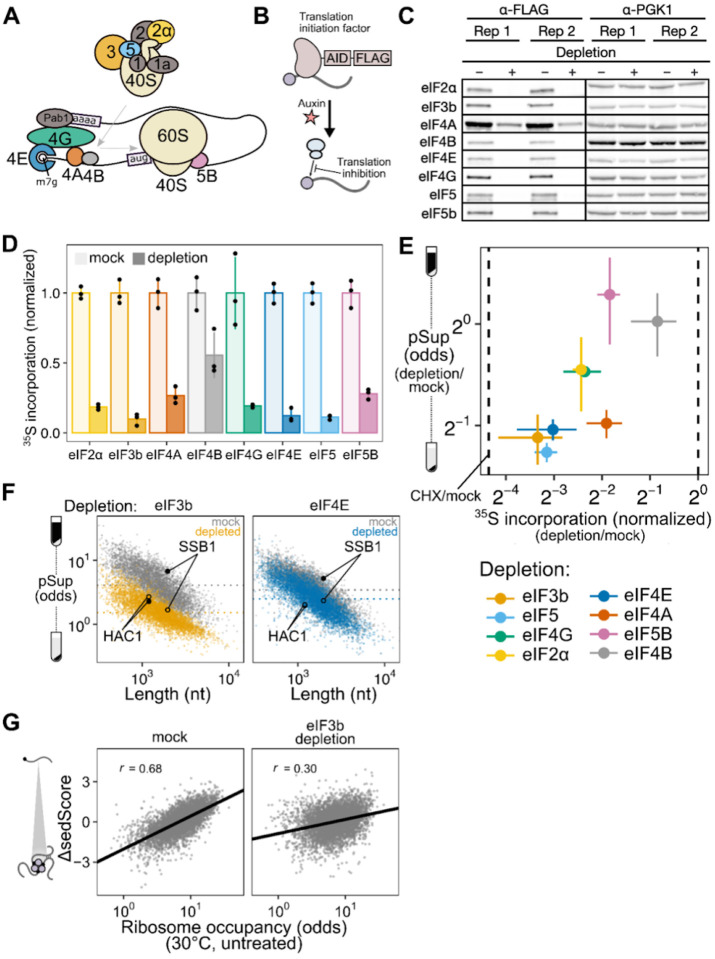
Global translational initiation inhibition triggers transcriptome-wide TIICs. (A) Translation initiation factors involved in various steps of initiation were (B) depleted via the auxin-inducible degradation system. (C) Depletion for each factor was verified via western blot with Pgk1 used as a control. (D) The effect on global translation level caused by each initiation factor was tested by measuring the incorporation of radiolabeled amino acids. Each depletion caused a drop in translation to varying amounts. (E) The pSup of the *PGK1* and *BEM2* transcripts is strongly related to the amount of translation block caused by each initiation factor depletion, suggesting that none of these factors are essential for condensation. (F) Sed-seq was used after elF3b and elF4E depletion to measure global sedimentation. Depletion of both factors, and especially elF3b, triggers global condensation—TIIC formation. (G) Left: The sedScore of transcripts correlates well with ribosome occupancy in the mock treated sample, but this association is attenuated after elF3b depletion.

To block translation initiation at multiple steps, we generated different yeast strains with auxin-inducible degron (AID) tags on eight factors acting at multiple stages of initiation (Figure 6A,B)^48,49^. Western blotting confirmed successful translation initiation factor degradation (Figure 6C), which resulted in polysome collapse (Figure S6A) and proteome-wide reduction in translation activity (Figure 6D, Figure S6B–D).

We used qPCR to quantify the average pSup of two transcripts, *PGK1* and *BEM2*, following two hours of initiation factor depletion. As predicted, blocking initiation triggered mRNA condensation, with the degree of translation initiation block correlating with the extent of resulting mRNA condensation (Figure 6E). Depletion of eIF4B and eIF5B caused negligible condensation, but also had the smallest effect on translation. By contrast, eIF4A depletion caused particularly strong mRNA condensation, consistent with previous evidence showing that eIF4A inhibition can trigger SG formation^59,60^.

How do initiation blocks affect condensation of individual transcript species? Our results above showed evidence for poorly initiated transcripts forming TIICs in unstressed cells, revealed by lower sedScore for transcripts with lower ribosome occupancy (Figure 5A). During global translation initiation block, we expect that all transcripts will form TIICs, leading to decreased pSups transcriptome-wide. To test this hypothesis, we performed Sed-seq on strains depleted for eIF4E, the mRNA cap-binding protein, and for eIF3b, the factor whose depletion led to the most severe block in translation. We observed transcriptome-scale mRNA condensation in both cases, to a profound degree after eIF3b depletion (Figure 6F). Because translationally repressed mRNAs already form TIICs in untreated cells, we predicted that they would show the smallest differences in sedimentation. Consistent with this prediction, initiation-inhibited *HAC1* mRNA showed almost no change after both depletions, whereas initiation-competent *SSB1* mRNA showed marked changes, reflecting the transcriptome average behavior (Figure 6F). Furthermore, reflecting the global convergence of sedimentation behavior during severe initiation block, the sedScores of transcripts in eIF3b-depleted cells are much less correlated with ribosome occupancy (Spearman *r* = 0.30) than the sedScores of transcripts in wild-type cells (Spearman *r* = 0.68) (Figure 6G).

Together, these results show that blocking translation initiation globally triggers global mRNA condensation and augments TIICs which are present in unstressed cells. We next sought to understand the relationship between TIICs, stress-induced mRNA condensation, and stress granules.

### TIICs are stress-granule precursors

**Figure 7:**
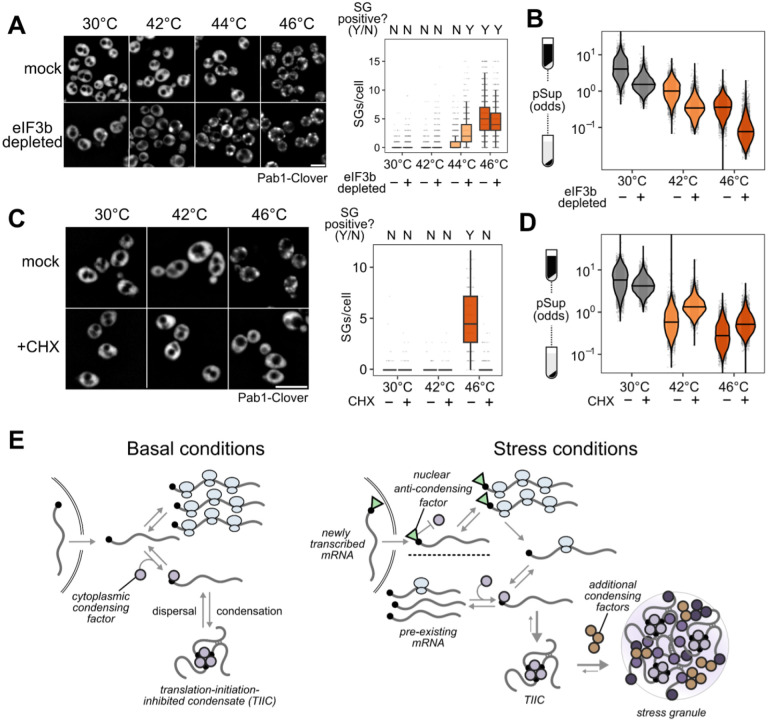
TIIC formation precedes and potentiates stress-granule formation. (A) Stress granules are potentiated in the elF3b depleted cells, shown by the earlier appearance of stress granules compared to the mock treatment. Right: Quantification of the presence of stress granules in all conditions. (B) Sed-Seq data comparing global condensation in elF3b depleted and mock cells after two hours of depletion followed by ten minutes of heat shock. elF3b depletion triggers more RNA condensation in each temperature. (C) Ten minutes of cycloheximide (CHX) treatment prior to stress prevents visible SG formation. (D) CHX treatment inhibits, but does not prevent stress-induced RNA condensation. (E) Model of the competition between translation initiation and TIIC formation during normal growth and stress. Well-translated transcripts are protected from condensation by competition between translation initiation and TIIC formation. During stress, newly transcribed transcripts escape stress-induced condensation, likely due to a 5’ bound protein or modification which inhibits condensation. In addition, global inhibition of translation leads to transcriptome-wide TIIC formation. These TIICs are precursors of visible stress granules, whose formation involves additional stress-induced condensing factors.

We counted stress granules before and after inhibiting translation initiation by eIF3b depletion, both in otherwise untreated and in heat-shocked cells (Figure 7A). Because automated counting scored some unstressed (30°C) cells as having multiple SGs, and all conditions show some degree of cell-to-cell variability, we scored populations of cells as SG-negative if the median number of SGs per cell was zero, and as SG-positive otherwise. Using this threshold, unstressed cells are SG-negative and cells shocked at 46°C are SG-positive (Figure 7A).

After eIF3b depletion at 30°C, which causes substantial transcriptome-wide mRNA condensation (Figure 7B), cells are SG-negative (Figure 7A). We conclude that inhibiting translation initiation by eIF3b depletion causes TIIC formation but not SG formation, further confirming the distinction between TIICs and SGs.

Upon heat shock at 44°C, otherwise untreated cells are SG-negative, but when eIF3b is depleted, cells become SG-positive (Figure 7A). Thus, eIF3b depletion potentiates SG formation, strongly suggesting that TIICs are the building blocks for stress granules.

In every case, heat stress amplifies the mRNA condensation induced by translation initiation depletion. As we have already established above, this cannot be attributed to translation inhibition alone. Instead, the obvious hypothesis is that stress triggers additional condensation processes. While we do not yet know which molecules are responsible for this additional stress-induced mRNA condensation, multiple RNA-binding proteins have already been shown to autonomously sense heat shock and undergo condensation^26,27,31,35^.

As a final test of the provisional conclusion that TIICs are building blocks for stress granules, we asked how pharmacologically blocking SG formation affects mRNA condensation. Treatment with cycloheximide (CHX) prior to stress prevents stress granule formation^31,61,62^, which we confirm—46°C heat-shocked cells are SG-positive, and 46°C heat-shocked cells pretreated with CHX are SG-negative (Figure 7C). There is a clear contrast between inhibiting translation initiation (via depletion of eIF3b) and inhibiting translation elongation (via CHX): the former triggers SGs, while the latter prevents SGs.

However, CHX does not block mRNA condensation; stress-induced condensation is reduced, but remains substantial (Figure 7D). These results mirror those from studies of stress-induced protein condensation^31^. We conclude that inhibiting SGs does not prevent mRNA condensation, consistent with our hypothesis that TIICs—condensed mRNAs—are precursors of stress granules.

## Discussion

What is the physiological role of mRNA condensation in and outside of stress? Which mRNAs condense during stress, and why? What is the relationship between mRNA condensation, its functional causes and consequences, and stress granule formation?

We find that, across multiple stress conditions, virtually all preexisting mRNAs form translationally silent condensates to a degree which depends on stress intensity. At the same time, stress-induced transcripts escape condensation and are robustly translated. These results echo important early observations that stress granules exclude bulk nascent mRNA^10,11^ and specific stress-induced heat shock protein transcripts^12,13^. Expanding and deepening these early results, our studies reveal that the timing of transcript production, rather than any particular transcript feature, is a primary determinant of escape from condensation; demonstrate the escape of dozens of stress-specific transcripts; and show that this escape from condensation guides selective translation. Still, a more fundamental result from our study, opening considerable new territory, is that stress granules per se play little if any role in these processes.

### Small mRNA condensates are pervasive in the absence of stress or stress granules

Using a range of approaches, we discover the existence of pervasive mRNA condensation in cells without stress granule formation, and even in the absence of any discernible stress. Our results illuminate a previously unreported level of molecular organization governed by translation initiation: initiation-blocked transcripts cluster into structures we term translation-initiation-inhibited condensates (TIICs). TIICs can be generated for specific mRNAs by blocking message-specific initiation, or at the transcriptome scale by blocking initiation at any of several stages; they do not require environmental stress for their formation; and they can form when stress granules are either absent or are pharmacologically blocked. This latter result mirrors the persistence of condensates of poly(A)-binding protein when stress granules are blocked^31^. In short, TIICs are not stress granules.

In our experiments, we make no attempt to isolate stress granules or their associated transcriptomes. Given that a range of stress conditions—physiological stresses such as 42°C heat shock and 5% ethanol, and the less-physiologically relevant but widely used 0.5% sodium azide—do not produce stress granules in our hands, but do produce considerable RNA condensation, considerable biology could be overlooked by focusing only on SG-forming conditions. We show that mRNA condensation, and specifically TIIC formation, precedes and potentiates stress granule formation, and we confirm by single-molecule FISH that stress-induced transcripts escape from stress granules. Overall, our results support a model in which stress-associated inhibition of translation initiation causes formation of TIICs which, under intense stress, further assemble into stress granules by separate processes.

### mRNA condensation in cells is not primarily driven by ribosome-free RNA

Stress granules have long been thought to form after translation inhibition and ribosome runoff, exposing ribosome-free RNA which serves as platform for new intermolecular interactions, whether directly between RNAs or mediated by RNA-binding proteins^36,37,63,64^. The profound effect of mRNA length in promoting apparent SG enrichment and in promoting RNA phase separation, with or without additional protein factors such as G3BP1, has provided a biophysical basis for the role of ribosome-free RNA: condensation of RNA due to multivalent RNA-mediated interactions would naturally be promoted by longer, and thus at least on average higher-valency, RNAs.

However, our results contradict the ribosome-free RNA model for condensation in multiple ways. First, we find that RNA length has little effect on stress-induced mRNA condensation once the effects of length on sedimentation, particularly for non-stress controls, are properly accounted for. Second, by comparing two abundant, similar-length, similarly ribosome-free mRNAs in budding yeast—*GCN4* and *HAC1*—we show that only one, *HAC1*, undergoes condensation. This condensation is reversed under conditions which release the *HAC1*-specific blockade in translation initiation, and synthetic versions of both mRNAs reproduce the behavior of these native transcripts. Third, the translation elongation inhibitor cycloheximide, which freezes ribosomes on mRNAs, blocks stress granule formation but does not block mRNA condensation. This latter result is particularly problematic for models of stress granules in which ribosome-free mRNA is required for earlier stages of mRNA condensation.

Specific proposals have suggested that condensation mediated by intermolecular RNA-RNA interactions will occur when the RNA chaperone capacity of cells is exceeded, such as during stress responses^64^. A prediction of this model is that formation of such condensates will not be possible in cells with ample RNA chaperone capacity. This prediction is contradicted by our results demonstrating the formation of TIICs of specific mRNA species, both *HAC1* and synthetic constructs, in otherwise unstressed cells, with no evidence of more widespread condensation. Notably, recent work suggests that a single stalled ribosome suffices to inhibit mRNA recruitment to stress granules^65^, consistent with a model in which ribosomes act as inhibitory signals for stress granule recruitment, rather than as a physical impediment to RNA-sequence-mediated recruitment. Such a model is entirely consistent with our results showing that CHX blocks stress granule formation but not TIIC formation.

Moreover, our data are inconsistent with phase separation of RNA. Phase separation of biological molecules occurs above a critical concentration, resulting in formation of a dense phase; many RNA granules are thought to form in this way^66^. We see no evidence for a critical concentration, either of specific RNAs or of bulk RNA. Indeed, higher-expression (and therefore higher-concentration) mRNAs are less likely to be found in TIICs (Figure S5A), and induction of transcripts correlates with their exclusion from condensates (Figure 2A), observations which go directly opposite the predictions of an RNA phase-separation model.

Altogether, we find no evidence supporting a role for ribosome-free RNA as a primary causal factor for mRNA condensation. Our results do not rule out an additional role for RNA-RNA interactions in stabilizing RNA-protein condensates once formed, which might explain some of the observed length-dependence in other datasets.

In contrast to length, we find a profound effect of translation initiation on condensation, supporting a model in which initiation and condensation compete. Such a model is conceptually similar to a translation-factor protection model proposed to regulate mRNA decay^67^. In essence, active translation initiation, focused on the 5’ end of the mRNA, physically blocks condensation, perhaps by blocking binding of a condensate-promoting factor (Figure 7E). Once initiated ribosomes proceed into the body of the message, the 5’ end is no longer blocked and condensation can proceed. The strong connection between initiation and condensation is another possible explanation for previously observed correlations between granule association and transcript length in well-controlled studies, as transcript length itself negatively correlates with initiation rate^47,50^.

What factors(s) cause condensation? While our data do not indicate a particular factor, they do appear to rule out a substantial number of potential individual candidates and narrow the search. Consider a provisional model in which a condensation factor targets (or is integral to) protein complexes formed at certain stages of initiation, such the model in mammalian systems in which stalled 48S preinitiation complexes serve as the seed of stress granules^68^. Depleting the condensation factor should inhibit condensation and solubilize mRNAs. Under this model, we can rule out initiation factors whose depletion promotes condensation (eIF2ɑ, eIF4E, eIF4G, eIF4A, eIF3b, eIF5) as condensation factors, and by the same logic, rule out direct binding of a condensation factor to these proteins. Moreover, these initiation factors regulate early steps in both mRNA activation (eIF4E/G/A = eIF4F) and 48S preinitiation complex formation (eIF2ɑ, eIF3b, eIF5), suggesting that neither associated protein complex either contains or is the target of a condensation factor.

Instead, our results are consistent with a model in which the condensation factor targets the mRNA cap directly, and scanning-complex assembly (eIF4F/mRNA/43S PIC) is the step at which the cap becomes blocked—thereby blocking condensation—until initiation is complete (Figure 7E). Disruption of either mRNA activation or 48S PIC formation blocks assembly of the scanning complex. Activation of mRNA by binding of eIF4F, including cap binding by eIF4E, is insufficient in this model to fully block cap if not followed by 43S recruitment, perhaps due to other proteins capable of destabilizing this interaction^69^.

The single case where we observe strong initiation inhibition with no apparent condensation, depletion of eIF5B (compare to similar inhibition by eIF4A depletion which induces substantial condensation), fits this model: eIF5B regulates an initiation step (60S subunit joining) which occurs after scanning complex assembly, such that its depletion disrupts initiation without exposing the cap and promoting TIIC formation^70^.

The cap-dependent condensation model naturally implicates other proteins which bind the cap, including nuclear cap-binding proteins and decapping proteins. Suppression of stress granules by preventing phosphorylation of the decapping protein Dcp2, a major component of P-bodies,^71^ hints at a potential role for the latter. Indeed, while TIICs differ from P-bodies in that their formation is not blocked by cycloheximide, they otherwise share the properties of being associated with poorly translated mRNAs and being precursors to stress granules^72^. Another similarity is that yeast P-body foci are not visible in the absence of stress, but oligomeric assemblies of P-body components are nevertheless detectable^73^. This raises the possibility that the TIICs we observe are associated with at least part of the complex interaction network that leads to P-body formation.

### How do newly synthesized mRNAs escape condensation?

Similar to the molecular determinants of TIIC formation, the specific determinants of escape from stress-induced condensation remain unknown. Our transcriptomic and reporter assays both show that transcripts transcribed during stress escape condensation regardless of sequence-encoded mRNA features or regulation by particular transcription factors. Consistent with our conclusion that timing is the key variable, an independent study of glucose withdrawal, another stress known to promote stress granule formation, also shows that expression timing, rather than sequence, determines whether mRNAs escape stress-induced translational repression^74^.

Timing of expression, in turn, suggests that new transcripts are marked in some way before or during nuclear export, and that this mark blocks condensation while permitting translation initiation. Translation is not required for exclusion of new transcripts, because even when translation is fully inhibited by depletion of eIF3b, newly transcribed transcripts still escape. What might this condensation-inhibiting mark be? Possibilities include an mRNA modification such as methylation (or its stress-induced absence), changes in polyadenylation, or addition or subtraction of a protein factor. Nuclear cap-binding proteins, for example, could be stabilized in the cytoplasm during stress instead of being exchanged during a pioneer round of translation. Indeed, prior work suggests these proteins can support active translation during stress^75^. Our study provides a range of new reagents which might be employed in the search for this putative anti-condensation mark. However, the main contribution of our study on this front is clarifying how the mark is made: not by mRNA features or promoters or transcription factors, but by when an mRNA is produced.

While timing plays an important role in regulating escape from condensation, during stress this effect is layered with the competition between translation initiation and condensation. We show that a pre-induced but well-translated *HSP26* reporter is better protected against condensation than a poorly-translated *PMU1* reporter. This effect is also seen transcriptome-wide, as even transcripts whose abundance decreases during stress escape condensation if they are well-translated. These results support the model above in which mRNA condensation is driven by a condensation factor whose binding is in competition with the initiation machinery.

### What are the functions of mRNA condensation?

In light of our results, an accounting of the cellular function of mRNA condensation must contend with three facts: the presence of condensation in unstressed cells, the strong causal link to translation initiation inhibition, and the exclusion of stress-induced messages.

The latter result argues strongly against any simple mRNA-feature-based biophysical model of RNA condensation, such as those invoking mRNA length, since we have shown that the timing of expression is decisive for mRNA recruitment. Exclusion of new messages and condensation of older messages also strongly favors an adaptive interpretation: stress-induced mRNA condensation helps cells rapidly redirect translational activity to transcripts most relevant to the cell’s current situation.

We hypothesize that mRNA condensation provides cells with useful regulatory control over the translationally active transcriptome through a simple mechanism: preventing reinitiation of ribosomes on translationally stalled mRNAs by sequestering their 5’ ends in a condensate. Condensation (which competes with decapping and potentially other processes in addition to reinitiation^67^) preserves these mRNAs for short-term retrieval by dispersal factors including molecular chaperones. Blocking reinitiation is crucial for redirecting translational activity, and separable from another effect which we do not explore but which is implied: protection of mRNAs from degradation^23,72,76^, which would otherwise be another mechanism to prevent reinitiation.

Stress enhances both effects of condensation, prevention of reinitiation and protection, through widespread inhibition of translation initiation and consequent TIIC formation, condensation of additional RNA-binding proteins and related factors. Chaperones responsible for dispersing TIICs under basal conditions are titrated away to these stress-induced condensates. Chaperone titration slows TIIC dispersal, keeping ribosomes free to initiate on the stream of uncondensed transcripts emerging from the nucleus, and thus focusing the cell’s translational activity on newly synthesized transcripts for an interval. This interval of translational focus ends when chaperones—whose genes, many under the transcriptional control of Hsf1, are powerfully induced by stress—become sufficiently abundant to disperse stress-induced condensates and TIICs back to pre-stress levels.

Multiple aspects of this condensation/dispersal model have been previously established: formation of reversible condensates during stress, many of which are stress-granule proteins^31,44,77^; colocalization of Hsf1-regulon chaperones with stress-induced condensates^2,78^; the requirement for these chaperones for efficient condensate dispersal in vivo and in vitro^2,79^; and titration of chaperones from inhibitory binding of Hsf1 to stress-induced substrates, activating transcription^80,81^. Here, we have uncovered the key regulatory steps linking translation initiation, condensation, and selective translation. Many testable predictions flow from the synthesis of these observations into a regulatory model, perhaps most importantly the potential chaperone-mediated aspects of dispersal under basal conditions and recovery, which we have not addressed here.

No part of this regulatory model requires formation of visible stress granules or any similar so-called membraneless organelles; small clusters which depend on chaperones for dispersal, as with poly(A)-binding protein, suffice. Our work here clarifies problems for understanding the function and formation of stress granules per se. What is the function, if any, of gathering smaller condensates into large cytosolic foci? How are stress granules built from TIICs and other stress-induced condensates? How does the presence of ribosomes on mRNA prevent stress granules without preventing mRNA condensation? To what extent are these separable stages in assembling TIICs, other protein and RNA condensates, and stress granules conserved over evolutionary time? Many of these remain grand challenges in stress granule biology^30^.

But separating mRNA condensation from stress granule formation is, in a sense, a smaller step than the other advance reported here: separating mRNA condensation from stress itself, and revealing a new layer of molecular organization in unstressed cells, one which extends even to the intensely studied central regulator of a major stress response. How TIICs form, dissolve, influence regulation, and so on outside of stress—how these previously unseen structures carry out previously unseeable activities—now must become a focus.

## Methods

### Cell growth and stress conditions

Unless otherwise noted, the BY4741 strain of Saccharomyces cerevisiae was used in experiments. All experiments were done with at least two biological replicates, starting from growth. Cells were grown at 30°C in synthetic complete dextrose media (SCD) for at least 12 hours to OD_600_ = 0.4 before being exposed to stress. Temperature stresses for sedimentation experiments were completed by centrifuging the culture and exposing the yeast pellet to either 42°C or 46°C water baths. Control cells were placed inside a 30°C incubator. Cycloheximide treated cells were pre-treated for 10 minutes with 100 μg/mL cycloheximide (Sigma #C7698–5G) before heat shock. Azide stresses were completed at either 0.5% w/v or 0.8% w/v for 30 min in SCD adjusted to pH 6.8 with NaOH. Azide was added from a 10% w/v sodium azide stock in water. Mock treatments were completed by adding pure water at the same volume to cultures. Ethanol stresses were completed by resuspending centrifuged cell pellets in SCD made with either 5%, 7.5%, 10%, or 15% ethanol for 15 min. Control cells were mock treated by resuspending in normal SCD. DTT treated cells were treated with 10 mM DTT for 15 minutes prior to harvesting. Temperature stresses for polysome sequencing and for tet-inducible reporter experiments were done by growing 250 mL of yeast in SCD overnight to OD_600_ = 0.4, collecting yeast via vacuum filtration onto a 0.45 μm filter (Cytiva 60206), putting the filter in 125 mL of pre-warmed media and incubating in a temperature controlled shaking water bath or incubator. After the indicated time, samples were harvested again via vacuum filtration and immediately scraped into liquid nitrogen.

Yeast transformations were performed either using a standard lithium acetate transformation or Zymo Frozen-EZ Yeast Transformation II Kit (Zymo #T2001) before plating on appropriate selection media^82^. Clones were verified by colony PCR and Sanger sequencing.

### Generation of spike-in RNA

In-vitro transcribed (IVT) RNA or purified *Schizosaccharomyces pombe* total RNA was used as spike-ins where noted. The IVT RNA was produced by first amplifying a linear DNA fragment encoding NanoLuc using Q5 polymerase (NEB #M0494S), and purifying the DNA using an NEB clean and concentrate kit. The RNA was then made using a T7 Highscribe kit (NEB #E2040S), treated with DNase I (NEB #M0303L) and purified using an NEB clean and concentrate kit (NEB #T2030).

For the *S. pombe* RNA, fission yeast (FY527) was grown in YES media (5 g/L yeast extract, 30 g/L glucose, 225 mg/L adenine, histidine, leucine, uracil and lysine hydrochloride) at 32°C until OD_600_ = 0.5, harvested by centrifugation (3 minutes at 2500 g), resuspended in Trizol, and lysed by vortexing with 0.5 mm zirconia glass beads before extracting RNA using Zymo Direct-zol kits (Zymo #R2072).

### Fractionation-by-Sedimentation-sequencing (Sed-seq)

Biochemical fractionation was completed similarly to Wallace et al.^31^, with the major exception that 20,000 g for 10 min was used rather than the original 100,000 g for 20 min. In short, 50 mL cultures of treated yeast were harvested by centrifugation at 3000 g for 5 minutes, then resuspended in 100 μL of soluble protein buffer (SPB: 20 mM HEPES, pH 7.4, 140 mM KCl, 2 mM EDTA, 0.1 mM TCEP, 1:200 protease inhibitor (Millipore #539136), 1:1000 SUPERase•In RNase Inhibitor (Invitrogen #AM2696)), and flash frozen in liquid nitrogen as a pellet in a 2 mL Eppendorf Safe-Lock tube (Eppendorf #0030123620) with a 7 mm steel ball (Retsch #05.368.0035). The cells were then lysed using a Retsch MM400 for 5×90s at 30 Hz, chilling in liquid nitrogen between each shaking repeat. The lysed cells were resuspended in 600 μL of SPB, and 100 μL of total sample was transferred to 300 μL of Trizol LS (Invitrogen #10296010). For the S. pombe spike-in experiment, purified S. pombe total RNA was added to the lysate immediately after resuspension in SPB. The remainder was centrifuged for 30 seconds at 3000 g, and 300 μL of clarified lysate was transferred to a new 1.5 mL tube. This was then centrifuged for 10 minutes at 20,000 g. A 100 μL supernatant sample was transferred to 300 μL of Trizol LS, and 400 μL of SPB was added to the pellet as a wash. After another spin at 20,000 g for 10 minutes, the supernatant was removed and the pellet was resuspended by vortexing for 15 minutes in 300 μL of Trizol LS and 100 μL of water. If required, 1 ng of spike-in transcript was added to each sample at this step before RNA was isolated using Zymo Direct-Zol RNA extraction columns (Zymo #R2052), and RNA integrity was assessed by the appearance of two sharp rRNA bands on a 1% agarose gel and quantified using the absorbance at 260 nm.

### RNA quantification by RT-qPCR

Reverse transcription for qPCR was either performed using gene-specific reverse priming with the iScript^™^ Select cDNA Synthesis Kit (Bio-Rad #1708897) or using NEB LunaScript RT SuperMix kit (NEB #E3010L). In both cases, manufacturer protocols were followed using an input of 2.5 ng of RNA per μL of reaction. For gene-specific priming, the reverse primer was used at 5 μM. The IDT Primetime gene expression master mix (IDT #1055771) was used for quantitative PCR on a Bio-Rad CFX384 instrument with Taqman probes (1.5 μM for primers; 600 nM probe). For samples with spike-ins, abundances were calculated relative to the spike-in abundance using the ΔΔCq method.

### Polysome collection and analysis

Around 100 mg of frozen yeast that was collected by vacuum filtration was transferred to a pre-chilled 2 ml Eppendorf “Safe-Lock” tube. Cells were lysed with a pre-chilled 7 mM stainless steel ball (Retsch #05.368.0035) by 5×90sx30Hz pulses in a Retsch MM100 mixer mill, chilling in liquid nitrogen (LN2) between pulses. Sample was resuspended in 10:1 (v/w) polysome lysis buffer (20 mM HEPES-KOH (pH 7.4), 100 mM KCl, 5 mM MgCl2, 200 μg/mL heparin (Sigma #H3149), 1% triton X-100, 0.5 mM TCEP (Goldbio #TCEP25), 100 μg/mL cycloheximide (Sigma #C7698–5G), 20 U/ml SUPERase•In (Invitrogen #AM2696), 1:200 Millipore protease inhibitor IV #539136). The lysate was clarified by centrifugation at 3000 g for 30 s, and the clarified lysate was transferred to new tube and aliquots were flash frozen in LN2.

A 10–50% continuous sucrose gradient in polysome gradient buffer (5 mM HEPES-KOH (pH 7.4), 140 mM KCl, 5 mM MgCl2, 100 μg/ml cycloheximide, 10 U/ml SUPERase•In, 0.5 mM TCEP) was prepared in SW 28.1 tubes (Seton #7042) using a Biocomp Gradient Master and allowed to cool to 4°C. Clarified lysate (200 μL) was loaded on top of the gradient, and gradients were spun in a SW28.1 rotor at 27,500 rpm for 3.5 hr at 4°C. Gradients were fractionated into 0.6mL fractions using a Biocomp Piston Gradient Fractionator with UV monitoring at 254 nm, and fractions were flash frozen in LN2. UV traces were normalized to the total signal starting with the 40S peak.

The samples were generated by pooling 50 μL of each fraction from the free fraction (before the monosome peak) and either separately pooling the fractions with 3+ ribosomes bound and the mono/di-some fractions (for the heat shock experiments), or by combining all ribosome-bound fractions together (azide and ethanol stresses). The spike-in (50 ng of S. pombe total RNA) was then added to each pooled sample. RNA was purified via ethanol precipitation (final concentrations of 0.3 M sodium acetate pH 5.2, 0.3 μg/mL glycoblue (Invitrogen #AM9516), and 70% ethanol) at −20°C overnight followed by centrifugation at 4°C for 30 minutes at 21,000 g. The pellet was washed with 1 mL of 70% ethanol before being resuspended in water. The purified RNA was then treated with Dnase I (NEB) before purifying again using an NEB RNA clean and concentrate kit (NEB #T2030).

### Sucrose cushion ribosome occupancy analysis

The ribosome occupancy (fraction of mRNA bound to ribosome) for the induction reporters was measured by spinning lysate through a sucrose cushion. Around 100 mg of frozen yeast was transferred to a pre-chilled 2 ml Eppendorf “Safe-Lok” tube. Cells were lysed with a pre-chilled 7 mM stainless steel ball (Retsch #05.368.0035) by 5×90sx30Hz pulses in a Retsch MM100 mixer mill, chilling in liquid nitrogen (LN2) between pulses. Sample was resuspended in 10:1 (v/w) polysome lysis buffer (20 mM HEPES-KOH (pH 7.4), 100 mM KCl, 5 mM MgCl2, 200 μg/mL heparin (Sigma #H3149), 1% triton X-100, 0.5 mM TCEP (Goldbio #TCEP25), 100 μg/mL cycloheximide (Sigma #C7698–5G), 20 U/ml SUPERase•In (Invitrogen #AM2696), 1:200 Millipore protease inhibitor IV #539136). The lysate was clarified by centrifugation at 3000 g for 30 s, and 500 μL clarified lysate was transferred to a new tube.

At this point the sample was split into +/− EDTA samples. For the +EDTA samples, 6 μL of 0.5 M EDTA (pH 8 in water) was added to 150 μL of clarified lysate and incubated on ice for 10 minutes. Then 100 μL of both samples (+/− EDTA) was gently added on top of 900 μL of matching sucrose cushion (5 mM HEPES-KOH (pH 7.4), 140 mM KCl, 5 mM MgCl2, 100 μg/ml cycloheximide, 10 U/ml SUPERase•In, 0.5 mM TCEP, 20% sucrose w/v, +/− 20 mM EDTA) and centrifuged for 60 minutes at 100,000 g in a TLA55 rotor (Beckman-Coulter) at 4°C. The top 250 μL of supernatant was removed as the supernatant sample and 100 μL of this was mixed with 300 μL Trizol LS. The remaining supernatant was discarded before resuspending the pellet in 100 μL water + 300 μL Trizol LS (pellet is 10x relative to supernatant). To the pellet 1 ng of spike-in RNA was added, but only 0.1 ng was added to the supernatant.

RNA was purified from the supernatant and pellet samples using Zymo Direct-Zol kits, then the abundances of target RNAs were quantified via qPCR as above. Ribosome occupancies were calculated by calculating the percentage of each transcript in the pellet, after correcting for the pelleting observed in the presence of EDTA (this separates EDTA-sensitive polysomes in the pellet from EDTA-insensitive condensates).

### RNA sequencing

In general, DNase I treated RNA was prepared for sequencing using rRNA depletion (Illumina RiboZero (Illumina #MRZY1306) or Qiagen FastSelect (Qiagen #334215) followed by NEB NEBNext Ultra II (NEB #E7760) or Illumina TruSeq library preparation and Illumina platform sequencing. Specific methods for library preparation, sequencing and initial data analysis are described below and the method used for each sample is indicated in Table S4.

### Sequencing analysis

#### Genome references

Saccharomyces cerevisiae reference genome files (S288C_reference_genome_R64-3-1_20210421) was downloaded from the Saccharomyces Genome Database (SGD)^83^. Schizosaccharomyces pombe reference genome files were downloaded from PomBase^84^. When appropriate (see Table S4), the sequences of the NanoLuc spike-in or the mCherry and Clover reporters were included in the genome and transcriptome files for mapping.

#### Group A (see Table S4):

Sequencing libraries were prepared by the University of Chicago Genomics Facility from DNase I treated RNA using Illumina RiboZero (Illumina #MRZY1306) and Illumina TruSeq library prep kits. Single end 50 bp sequencing was performed on an Illumina HiSeq 4000 sequencer.

Sequencing reads were trimmed using TrimGalore (v0.6.10, https://github.com/FelixKrueger/TrimGalore) using default settings (e.g. trim_galore --gzip --fastqc_args ‘--outdir fastqc/’ -j 4 -o trimmed --basename FW32 EW_FW32_R1.fastq.gz). They were mapped using STAR v2.7.10 b^85^ (e.g. STAR --outSAMtype BAM Unsorted --readFilesCommand gunzip -c --sjdbGTFfile saccharomyces_cerevisiae_R64-3-1_20210421_nofasta_geneid.gff --sjdbGTFtagExonParentTranscript Parent --sjdbGTFfeatureExon CDS -- sjdbGTFtagExonParentGene gene_id --runThreadN 4 --alignMatesGapMax 20000 --limitBAMsortRAM 1445804817 --genomeDir STAR_saccharomyces_cerevisiae_R64-3-1_20210421_allchrom --outFileNamePrefix mapped_reads/FW32/FW32_ --readFilesIn trimmed/FW32_trimmed.fq.gz). To generate estimated counts and transcript per million (TPM) values, sequencing reads were mapped to the yeast transcriptome using kallisto v0.48.0^86^ (e.g. kallisto quant -i Scerevisiae_orf_coding_all_Scerevisiae_rna_coding.fasta.idx -o kallisto_quant/FW32 --single -l 200 -s 1 --rf-stranded --bootstrap-samples=50 -t 1 trimmed/FW32_trimmed.fq.gz).

#### Group B (see Table S4):

Sequencing libraries were prepared by from DNase I treated RNA using Qiagen FastSelect (Qiagen #334215), NEBNext Multiplex Oligos (UMI Adaptor RNA Set 1, NEB #E7335L) and NEBnext Ultra II Directional RNA library prep kits (NEB #E7760L). Paired end 200 bp sequencing with additional reads for dual 8/8 indices plus the 11nt UMI after the i7 index was performed on an Illumina NovaSeq 6000 at the University of Chicago Genomics Facility.

The unique molecular indices (UMIs) were extracted from fastq R2 using Umi-Tools v1.1. 4^87^ and stored in fastq R1 and R3 (e.g. umi_tools extract-- bc-pattern=XXXXXXXXNNNNNNNNNNN -I AD-JB-1S-HG02_S2_R2_001.fastq.gz -- read2-in=AD-JB-1S-HG02_S2_R1_001.fastq.gz -- read2-out=labeled_fastq/HG002/HG002_R1.umi.fastq. Sequencing reads were then trimmed using TrimGalore (v0.6.10, https://github.com/FelixKrueger/TrimGalore) using default settings (e.g. trim_galore --paired --gzip --fastqc_args ‘--outdir fastqc/’ -j 4 -o trimmed --basename HG002 labeled_fastq/HG002/HG002_R1.umi.fastq labeled_fastq/HG002/HG002_R3.umi.fastq). They were mapped using STAR v2.7.10 b^85^ (e.g. STAR --outSAMtype BAM Unsorted --readFilesCommand gunzip -c --sjdbGTFfile spike_saccharomyces_cerevisiae_R64-3-1_20210421_geneid.gff3 -- sjdbGTFtagExonParentTranscript Parent --sjdbGTFfeatureExon CDS -- sjdbGTFtagExonParentGene gene_id --runThreadN 4 --alignMatesGapMax 20000 -- limitBAMsortRAM 1445804817 --genomeDir STAR_spike_saccharomyces_cerevisiae_R64-3-1_20210421 --outFileNamePrefix mapped_reads/HG002/HG002_ --readFilesIn trimmed/HG002_val_1.fq.gz trimmed/HG002_val_2.fq.gz). Umi-Tools was then used again to deduplicate the reads (e.g. umi_tools dedup --stdin=mapped_reads/HG002/HG002_Aligned_Sorted.out.bam -- chimeric-pairs=discard --unpaired-reads=discard --spliced-is-unique --paired -S mapped_reads/HG002/HG002_Aligned.sortedByCoord.dedup.out.bam). The reads were split again into fastq files using samtools v1.16.1^88^, and then estimated counts and TPMs were generated using kallisto v0.48. 0^86^ (e.g. kallisto quant -i spike_Scerevisiae_orf_coding_all_Scerevisiae_rna_coding.fasta.idx -o kallisto_quant/HG002 -- rf-stranded --bootstrap-samples=50 -t 1 mapped_reads/HG002/HG002_Aligned_dedup_R1.fastq.gz mapped_reads/HG002/HG002_Aligned_dedup_R3.fastq.gz).

#### Group C (see Table S4):

Sequencing libraries were prepared by the University of Chicago Genomics Facility from DNase I treated RNA using Qiagen FastSelect (Qiagen #334215) and Illumina Stranded mRNA Prep (Illumina #20020595) kits. Paired end 200 bp sequencing was performed on an Illumina NovaSeq 6000.

Sequencing reads were trimmed using TrimGalore (v0.6.10, https://github.com/FelixKrueger/TrimGalore) using default settings (e.g. trim_galore --paired --fastqc_args ‘--outdir fastqc/’ -j 4 -o trimmed --basename F02 AD-JB-F02_S44_R1_001.fastq.gz AD-JB-F02_S44_R2_001.fastq.gz). They were mapped using STAR v2.7.10b^85^ (e.g. STAR --outSAMtype BAM Unsorted --readFilesCommand gunzip -c --sjdbGTFfile spike_saccharomyces_cerevisiae_R64-3-1_20210421_geneid.gff3 -- sjdbGTFtagExonParentTranscript Parent --sjdbGTFfeatureExon CDS -- sjdbGTFtagExonParentGene gene_id --runThreadN 4 --alignMatesGapMax 20000 -- limitBAMsortRAM 1445804817 --genomeDir STAR_spike_saccharomyces_cerevisiae_R64-3-1_20210421 --outFileNamePrefix mapped_reads/F02/F02_ --readFilesIn trimmed/F02_val_1.fq.gz trimmed/F02_val_2.fq.gz). The estimated counts and TPMs were generated using kallisto v0.48.0^86^ (e.g. kallisto quant -i spike_Scerevisiae_orf_coding_all_Scerevisiae_rna_coding.fasta.idx -o kallisto_quant/F02 -- fr-stranded --bootstrap-samples=50 -t 1 trimmed/F02_val_1.fq.gz trimmed/F02_val_2.fq.gz).

### Calculation of pSup

Public code for calculating pSup from sequencing data is available here: https://github.com/jabard89/sedseqquant. The statistical model used to estimate the proportion in supernatant (pSup) was based on that used in Wallace et al. (2015)^25^. For each fractionated sample, the number of counts of mRNA within each fraction—total (T), supernatant (S), and pellet (P)—were extracted from RNA-sequencing data (see [“Sequencing Analysis” section above]). While mRNAs are expected to obey conservation of mass in the original fractionated lysate (*T*_*i*_ = *S*_*i*_ + *P*_*i*_ for mRNA species i), this assumption does not hold in the ratios of abundances directly inferred from the data. Instead, for a particular experiment, *T*_*i*_ = α_*S*_ *S*_*i*_ + α_*P*_ *P*_*i*_ where we refer to the per-experiment constants α_*S*_ and α_*P*_ as mixing ratios which reflect differential processing and measurement of individual fractions. In order to estimate mixing ratios, and thus recover the original stoichiometry, we assume conservation of mass for each mRNA in the sample, and then estimate the mixing ratios under this constraint using a Bayesian model^89^. We assume negative binomial noise for each count measurement, and log-normal underlying distribution of mRNA abundance. Specifically, we model counts as follows:

$$log\left(T_{i}\right)\sim NB\left(log\left(\alpha_{S}S_{i}+\alpha_{P}P_{i}\right),\phi \right)$$

where

*T*_*i*_ = measured abundance of mRNA *i*,
*S*_*i*_ = measured abundance in supernatant of mRNA *i*,
*P*_*i*_ = measured abundance in pellet of mRNA *i*,
α_*S*_ = mixing ratio of supernatant sample,
α_*P*_ = mixing ratio of pellet sample

With the following priors:

α_*S*_ ~ Γ(1, 1)
α_*P*_ ~ Γ(1, 1)
σ ~*Cauchy* (0, 3)

We implemented the model above in R using the probabilistic programming language STAN, accessed using the rstan package^90,91^ and used all mRNA with *counts* > 20 to estimate mixing ratios for each sample. These mixing ratios were then used to calculate the pSup for mRNA $\text{i:}\;pSup_{i}=\frac{\alpha_{S}S_{i}}{\alpha_{S}S_{i}+\alpha_{P}P_{i}}$.

### Other bioinformatic analyses

#### Transcript features

Transcript features were extracted from Saccharomyces Genome Database (SGD)(Cherry et al. 2012). Targets of HSF1 and MSN2/4 were based off those reported in Pincus et al. 2018^40^ and Solis et al. 2016^41^. Transcript UTR lengths were taken as the median value reported by long read transcript sequencing in Pelechano et al. 2013^92^, or, when no data was reported, the median UTR length in yeast was used as the default. Pombe transcript lengths, including the lengths of the UTRs, was taken from PomBase^84^.

#### Transcript abundance

The transcript abundance is reported as the geometric mean of the TPM value for two biological replicates, estimated by kallisto analysis of the Total fraction for each sample. Changes in transcript abundance were calculated using DeSeq2^93^.

#### sedScore calculation

In order to calculate sedScores, the pSup for each transcript was converted to a log-odds scale, and transcripts were arranged by their length (including UTRs), and then binned into groups of 100. For each transcript in the bin, the standard deviation from the mean within the bin was used to calculate a Z-score. Individual Z-scores from two biological replicates were calculated and then averaged together for the final reported sedScore.

#### Ribosome occupancy

Because Polysome-seq data was collected with spike-in values for each fraction (Total, Free, Mono/Poly), it is possible to calculate the absolute ribosome occupancy (% of a transcript which is bound to at least one ribosome) for each transcript. This value is calculated by normalizing transcript abundance for each fraction (TPMs output by kallisto) to the median abundance of the spike-in transcripts. All S. pombe spike-in transcripts with more than 100 estimated counts were used to calculate the spike-in abundance. The ribosome occupancy is then calculated as abundance_bound_/(abundance_bound_ + abundance_free)_.

#### Ribosome association

In stressed samples, it is possible that condensed RNA pellets to the bottom of the sucrose gradient, making it difficult to calculate the absolute ribosome occupancy. Thus, for stressed samples, we calculate a “ribosome association” score which is TPM_rib. bound_/TPM_Total_^94^. This metric is similar to “translation efficiency” scores calculated for ribosome profiling studies^50^. The change in ribosome association upon stress was calculated using DeSeq2^93^, similar to reported methods for calculating changes in translation efficiency using DeSeq2^95^.

#### RNA structure analysis

The sequence for the 5′ UTR + the first 20 nucleotides of the CDS was extracted using the 5′ UTR lengths described above from Pelechano et al. 2013^92^. The folding energy for each UTR was then calculated using RNAFold from the ViennaRNA package^96^. Because the folding energy correlates directly with length, a normalized structure score was calculated for each transcript by dividing the calculated folding free energy by the length of the UTR.

### Induction reporters

Reporters for pulsed induction were generated by Gibson assembly of gene fragments with a TET-inducible promoter designed for tight control of induction levels^97^. Assembly pieces were derived either from gene fragments ordered from IDT or Twist Biosciences or from PCR amplification of other plasmids. Fragments were assembled into backbones generated by golden gate cloning using protocols and plasmids from the Yeast Toolkit^98^, and the plasmids were sequenced by overlapped Sanger sequencing. Plasmids were linearized with NotI prior to transformation.

The PMU1 reporter contains the 5′ UTR and 3′ UTR of the native PMU1 gene and the CDS is a fusion of the PMU1 CDS with nanoluciferase-PEST^99^. The HSP26 reporter contains the 5′ UTR and 3′ UTR of the native HSP26 gene, but the CDS is a fusion of the TPI1 CDS and nanoluciferase-PEST. The TPI1 fusion was used to avoid potential artifacts caused by a large pre-induction of HSP26 molecular chaperone and because TPI1 is well translated during stress and of a similar length (645 nt for HSP26 vs 745 nt for TPI1). Reporters were integrated at the HO locus using hygromycin selection in a strain of yeast containing a C-terminal auxin tag on Sui2, along with the inducible TIR1 ligase at the LEU locus, and the TetR protein at the his locus (see Table S1 for full genotype).

For induction of reporters concurrently with stress, 1 μM anhydrotetracycline (aTC, Cayman #CAYM-10009542–500) was added from a 10 mM stock prepared in DMSO at the beginning of the stress. For pre-induced samples, 0.1 μM aTC was added to yeast in SCD at OD_600_ = 0.2 and samples were incubated at 30°C for 45 minutes. Samples were then either washed 3x with SCD via centrifugation, or 1x via vacuum filtration before resuspending in prewarmed SCD. Stress was then initiated 30 minutes after washing had begun to ensure complete shutoff of reporter transcription. Samples were then fractionated as described above either using the Sed-Seq protocol to calculate pSup or the sucrose cushion fractionation to calculate ribosome occupancy.

### Engineering solubility reporters

Solubility reporters were engineered using the Yeast Toolkit [Lee et al., 2015] (see Table S1 and S2). Variable 5′UTRs were engineered depending on the construct and genetically integrated in front of two copies of Clover, all driven by the constitutive TPI1 promoter and with the TPI1 3′ UTR. Each reporter construct also had a copy of mCherry with a TPI1 promoter, 5′UTR and 3′UTR. This construct was inserted into the Leu2 locus with leucine selection.

Steady state protein levels were measured using flow cytometry by normalizing the Clover signal to the mCherry signal in each cell. Data was analyzed with a custom script using FlowCytometryTools in python and then exported and plotted in R. The standard Sed-seq protocol was used to measure the condensation behavior of each strain. Steady state mRNA levels were extracted from the Total sample of the Sed-seq experiment and translation efficiency was calculated as the steady state protein level divided by steady state RNA level.

### Auxin-mediated depletions

Auxin induced degron depletions were adapted from the approach in Mendoza-Ochoa et al. [2019]. In short, the endogenous protein of interest was genetically engineered to contain the degron tag in a strain of yeast in which a β-estradiol inducible TIR1 ligase had been genetically integrated at the LEU locus. Some of the strains contained the original Oryza sativa TIR1 (OsTIR1), while others used a variant engineered for more specificity OsTIR1(F74G)^49^ as indicated in Table S1. The auxin-FLAG degrons were installed at either the 5´ or 3´ end of genes using CRISPR plasmids from the yeast toolkit. A PCR-generated DNA template was co-transformed with a Cas9 and gRNA containing URA3 selectable plasmid as previously described^98,100^. The CRISPR integrations were verified by PCR and Sanger sequencing and the URA3 plasmid was removed by selecting for colonies which did not grow on URA plates.

For depletion experiments, yeast were grown at 30°C in YPD to OD_600_ = 0.1. To induce TIR1 ligase, 5 μM β-estradiol (10 mM stock in DMSO) or an equivalent volume of DMSO (for mock treatment) was added to each culture and they were incubated for 75 minutes. To induce degradation, either 100 μM of Indole-3-acetic acid sodium salt (Sigma #I5148, 250 mM stock in DMSO) or 5 μM of 5-Ph-IAA (Medchemexpress #HY-134653, 5 mM stock in DMSO) was added. After 2 hours of auxin exposure, cells were temperature treated and then harvested and fractionated as normal.

### Radiolabeling quantification of translation

Yeast cells were cultured overnight in YPD until they reached an OD_600_ = 0.1. Auxin-inducible yeast strains were then treated with beta-estradiol and auxin, as detailed above, then translation was measured following a published protoco l^101^. After a 1.5-hour depletion period, 1 mL of sample was transferred to 1.5mL tubes, then 1 μCi/mL of mixed 35 S-L-methionine and 35S-L-cysteine media were added to each sample (Perkin-Elmer EasyTag #NEG772002MC). Samples were incubated for 30 minutes at 30°C with shaking (15 minutes for heat shocks), then cells were treated with 200 μL of 50% trichloroacetic acid (TCA), chilled on ice for 10 minutes, heated at 70°C for 20 minutes, and cooled again for 10 minutes. The samples were subsequently collected on glass microfiber filters (Sigma #WHA1823025) loaded onto a vacuum manifold (Millipore #XX2702550), washed with 3× 5 mL 5% TCA and 2× 5mL 95% ethanol, and air-dried for at least 12 hours at room temperature. Filters were then immersed in scintillation fluid (Perkin Elmer #6013179), and radioactivity levels were quantified in “counts per minute” through liquid scintillation counting on a Tri-Carb machine.

### Western blotting

Western blots were performed as described in a published protocol^102^. For each sample, 1mL of yeast culture was spun down at 2500 g for 2 minutes, and the pellet was resuspended in 50 μL of 100 mM NaOH. The samples were incubated for 5 minutes at RT, spun at 20,000g for 1min, and resuspended in 50 μL of 1x Laemmli buffer (Bio-rad #1610737) with 5% β-mercaptoethanol. Samples were then boiled for 3 minutes, clarified at 20,000 g for 2 minutes and 15 μL was loaded onto a 4–20% tris-glycine SDS-PAGE gel (Biorad #5671094). Proteins were then transferred to nitrocellulose (Sigma #10600001) using a wet transfer apparatus (Bio-rad #1704070). The membrane was blocked for 1 hour with 5% milk in TBST buffer, then incubated rocking overnight at 4°C with 1:3000 dilution of anti-FLAG antibody (Sigma #F1804) and 1:10,000 dilution of anti-PGK1 antibody (Invitrogen #459250) in 5% milk solution. Westerns were visualized using 1:20,000 dilutions of fluorophore conjugated secondaries (Licor #926–32212 and #925–68073) and visualized on a Licor Odyssey CLx. Band intensities were quantified in ImageJ and normalized to PGK1 signal.

### Fluorescence microscopy and stress granule quantification

Standard confocal microscopy was completed as in Wallace et al. [2015], generally using Pab1-Clover as the SG marker unless otherwise noted. Cells were grown to log-phase as previously described. 1mL of cells were transferred to 1.5mL Eppendorf tubes. For heat stress, cells were shocked in a heat block, spun down in a microfuge, and 950 uL of supernatant were removed. For azide stress, 10% (w/v) azide or water was added directly to the 1mL of cells to proper dilution of aizde. For ethanol stress, cells were spun down in microfuge and resuspended in media with appropriate amounts of ethanol. 1.5 uL of treated cells were then placed on a glass slide and imaged immediately. For AID treatment, cells were treated as previously described, and were imaged immediately after a 2 hour exposure to Auxin. For cycloheximide treatment, cells were exposed to 100 ug/mL of cycloheximide for 10 minutes, stressed for 10 minutes, and then imaged immediately. Cells were imaged on an Olympus DSU spinning disc confocal microscope using a 100× 1.45 TIFM oil objective (PlanApo) and the FITC filter cube for the Clover fluorophore in Z-stacks. Representative images are maximum projections of the collected z-stacks. Maximum projection images of the cells were used to quantify the number of stress granules per cell using CellProfiler.

### Single-molecule fluorescence in situ hybridization (smFISH)

Custom Stellaris^®^ RNA FISH Probes were designed against SSB1, SSA4, HSP104, and ADD66 by utilizing the Stellaris^®^ RNA FISH Probe Designer (Biosearch Technologies, Inc., Petaluma, CA) available online at www.biosearchtech.com/stellarisdesigner (Table S3). Each Stellaris FISH Probe set was labeled with Quasar670 (Biosearch Technologies, Inc.). smFISH was done as previously described^103,104^. Yeast cultures were grown to an OD of 0.3–0.4 in SCD, spun down at 3k g for 3 min. Cells were then suspended into 4mL of culture and Oregon Green HaloTag reagent (Promega #G2801) was added to a final concentration of 2uM. Cells were then resuspended and split into final cultures of 25 mL. Cells were then spun again at 3000g for 3min, and 23mL were removed, such that 2mL of media remained. Cells were then stressed as stated before. 19.85mL of pre-warmed media was then added to each falcon tube, and 3.15 mL of 4% paraformaldehyde (Electron Microscopy Services #15714) was immediately added. Cells were incubated at room temperature for 45 min at room temperature, gently rocking. Cells were spun down at 4°C and washed with ice-cold buffer B. Cells were resuspended into 1mL of Buffer B (1.2M sorbitol, 100mM KHPO4, pH = 7.5) then transferred to a 12-well plate. Cells were crosslinked in a Spectrolinker UV Crosslinker at a wavelength of 254nm by exposure to 100 mJ/cm^2 twice with 1 min break in between^105^. Cells were pelleted for 3min at 2000rpm and then resuspended into spheroplast buffer (1.2 M sorbitol, 100 mM KHPO4, pH = 7.5, 20mM ribonucleoside-vanadyl complex (NEB # S1402S), 20 mM B-mercaptoethanol). 25U/OD of lyticase (Sigma #L2524–10KU) were added to each sample. Cell digestion was performed at 30°C and was monitored using a benchtop phase contrast microscope, such that cells were about 50%−70% digested. Digestion was stopped by spinning cells at 4°C for 3min at 2000 rpm and two washes twice in ice cold buffer B and resuspended in 1mL Buffer B. 250 uL of cells were placed onto a poly-L lysine coated coverslip and incubated at 4C for 1hr. Cells were washed with 2 mL of Buffer B and then stored in ice-cold 70% ethanol for at least 3 hours. Coverslips were rehydrated in 2 xSSC and then washed twice in pre-hybridization buffer (2x SSC + 5% formamide (Sigma #344206–100ML-M)) for 5 minutes each. smFISH probes were concurrently prepared. A mixture of 0.125uL of 25uM smFISH probes, and 2.5uL of 10mg/ml yeast tRNA (Thermo #AM7119) and 2.5uL of 10mg/mL salmon sperm DNA was dehydrated in a Speedvac at 45°C. The dried pellet was rehydrated was resuspended in 25 μl hybridization mix (10% formamide, 2×SSC, 1mg/mL BSA, 10 mM Ribonucleoside–vanadyl complex (Thermo #15632011) and 5 mM NaHPO4, pH 7.5) and boiled at 95 °C for 2 min. 18uL of resuspended probes were spotted onto a piece of Parafilm and coverslips were placed cell-side down into hybridization mixture. Hybridization occurred at 37°C for 3 hours. Coverslips were then washed at 37°C for 15min in 2x SSC + 5% formamide, then in 2x SSC buffer, then 1xSSC buffer. They were then submerged in 100% Ethanol, dried, and then mounted into ProLong Gold antifade with DAPI (Thermo P36941).

### smFISH image acquisition and analysis

smFISH images were taken on a Nikon TiE microscope with a CFI HP TIRF objective (100x, NA 1.49, Nikon), and an EMCCD (Andor, iXon Ultra 888). Nikon TiE epifluorescent microscope. Samples were excited using the 647nm laser (Cobolt MLD) (~15–20 mW for 200–300ms), poly-A FISH was imaged using the 561nm laser (Coherent Obis) (~15–20 mW for 200–300ms), and Pab1-Halotag signal was imaged with a 488nm laser (Cobolt MLD) (~10–15 mW for 200–300 ms), and DAPI (CL2000, Crystal Laser) (~5–10 mW for 100 ms). Imaging of the nucleus was done using the 405nm laser and DIC images were taken as well. Z-stacks of 21 planes, 2uM thick were obtained. Images were analyzed using FISH-quant^106^. Briefly, RNA spots were identified using big fish. For the smFISH colocalization analysis, RNA spot intensities were normalized by dividing by the mean intensity of each cell. For each RNA spot, the mean Pab1 intensity in a 3×3 pixel square around the centroid was calculated. The Pab1 intensity was then measured for 100 random locations in the cell in 3×3 pixel locations. Finally, a distribution was calculated for both the random Pab1 signal and the Pab1 signal that corresponds to a RNA spot. The Z-score of the mean intensity of the Pab1 signal in a RNA spot compared to the Pab1 signal in a random spot was compared, and this is termed the ‘colocalization score’. Each Z-score is calculated independently for each cell, and the average shown is for every cell.

### Simulation of mRNA condensation

The underlying biophysical model for pSup in the absence of condensation is $\text{pSup}(g)=1-\beta L_{g}^{\chi }$ for a mRNA transcript encoded by gene *g*, of length *L*_*g*_. In conditions where there is mRNA condensation, governed by parameter μ per-transcript and ν per-nucleotide, the model is: $\text{pSup}(g)=\left(1-\beta L_{g}^{\chi }\right)e^{-\left(\mu +vL_{g}\right)}$. These models were fitted to sedimentation on the log-odds(pSup) scale, i.e. approximating the log-odds sedScore as normally distributed. Non-linear least squares fits were performed using the nls function in R. See supplemental text for details.

### Statistical analyses

Unless otherwise stated, all experiments were performed as at least two biological replicates, and the mean or geometric mean value (for log-distributed transcript abundance data) was calculated from the replicates. Unless otherwise noted, all correlation values are reported as Spearman’s rank correlation coefficient and significance tests comparing groups of data points were performed using a Wilcoxon rank-sum test, with a Bonferroni correction when multiple groups were being compared (*P < 0.05, **P < 0.01, ***P < 0.001. ‘N.S.’ denotes not significant (P ≥ 0.05).

## Supplementary Material

Supplement 1

## Data Availability

All raw sequencing data generated for this project have been deposited in GEO under accession code GSE265963. All other data and code is deposited at https://github.com/jabard89/RNA_Condensation_2024/ or available upon request.

## References

[1] Farny N.G., Kedersha N.L., and Silver P.A. (2009). Metazoan stress granule assembly is mediated by P-eIF2alpha-dependent and -independent mechanisms. RNA 15, 1814–1821. 19661161 10.1261/rna.1684009 PMC2743051

[2] Cherkasov V., Hofmann S., Druffel-Augustin S., Mogk A., Tyedmers J., Stoecklin G., and Bukau B. (2013). Coordination of translational control and protein homeostasis during severe heat stress. Curr. Biol. 23, 2452–2462. 24291094 10.1016/j.cub.2013.09.058

[3] Hoyle N.P., Castelli L.M., Campbell S.G., Holmes L.E.A., and Ashe M.P. (2007). Stress-dependent relocalization of translationally primed mRNPs to cytoplasmic granules that are kinetically and spatially distinct from P-bodies. J. Cell Biol. 179, 65–74. 17908917 10.1083/jcb.200707010 PMC2064737

[4] Khong A., Matheny T., Jain S., Mitchell S.F., Wheeler J.R., and Parker R. (2017). The Stress Granule Transcriptome Reveals Principles of mRNA Accumulation in Stress Granules. Mol. Cell 68, 808–820.e5. 29129640 10.1016/j.molcel.2017.10.015 PMC5728175

[5] Protter D.S.W., and Parker R. (2016). Principles and Properties of Stress Granules. Trends Cell Biol. 26, 668–679. 27289443 10.1016/j.tcb.2016.05.004 PMC4993645

[6] Nover L., Scharf K.D., and Neumann D. (1989). Cytoplasmic heat shock granules are formed from precursor particles and are associated with a specific set of mRNAs. Mol. Cell. Biol. 9, 1298–1308. 2725500 10.1128/mcb.9.3.1298-1308.1989 PMC362722

[7] Riback J.A., Katanski C.D., Kear-Scott J.L., Pilipenko E.V., Rojek A.E., Sosnick T.R., and Drummond D.A. (2017). Stress-Triggered Phase Separation Is an Adaptive, Evolutionarily Tuned Response. Cell 168, 1028–1040.e19. 28283059 10.1016/j.cell.2017.02.027 PMC5401687

[8] Banani S.F., Lee H.O., Hyman A.A., and Rosen M.K. (2017). Biomolecular condensates: organizers of cellular biochemistry. Nat. Rev. Mol. Cell Biol. 10.1038/nrm.2017.7. PMC7434221 28225081

[9] Mittag T., and Pappu R.V. (2022). A conceptual framework for understanding phase separation and addressing open questions and challenges. Mol. Cell. 10.1016/j.molcel.2022.05.018. PMC9233049 35675815

[10] Collier N.C., Heuser J., Levy M.A., and Schlesinger M.J. (1988). Ultrastructural and biochemical analysis of the stress granule in chicken embryo fibroblasts. J. Cell Biol. 106, 1131–1139. 3283146 10.1083/jcb.106.4.1131 PMC2114993

[11] Kedersha N.L., Gupta M., Li W., Miller I., and Anderson P. (1999). RNA-binding proteins TIA-1 and TIAR link the phosphorylation of eIF-2 alpha to the assembly of mammalian stress granules. J. Cell Biol. 147, 1431–1442. 10613902 10.1083/jcb.147.7.1431 PMC2174242

[12] Kedersha N., and Anderson P. (2002). Stress granules: sites of mRNA triage that regulate mRNA stability and translatability. Biochem. Soc. Trans. 30, 963–969. 12440955 10.1042/bst0300963

[13] Stöhr N., Lederer M., Reinke C., Meyer S., Hatzfeld M., Singer R.H., and Hüttelmaier S. (2006). ZBP1 regulates mRNA stability during cellular stress. J. Cell Biol. 175, 527–534. 17101699 10.1083/jcb.200608071 PMC2064588

[14] Grousl T., Ivanov P., Frydlova I., Vasicova P., Janda F., Vojtova J., Malinska K., Malcova I., Novakova L., Janoskova D., (2009). Robust heat shock induces eIF2alpha-phosphorylation-independent assembly of stress granules containing eIF3 and 40 S ribosomal subunits in budding yeast, Saccharomyces cerevisiae. J. Cell Sci. 122, 2078–2088. 19470581 10.1242/jcs.045104

[15] Bounedjah O., Desforges B., Wu T.-D., Pioche-Durieu C., Marco S., Hamon L., Curmi P.A., Guerquin-Kern J.-L., Piétrement O., and Pastré D. (2014). Free mRNA in excess upon polysome dissociation is a scaffold for protein multimerization to form stress granules. Nucleic Acids Res. 42, 8678–8691. 25013173 10.1093/nar/gku582 PMC4117795

[16] Hofmann S., Kedersha N., Anderson P., and Ivanov P. (2020). Molecular mechanisms of stress granule assembly and disassembly. Biochim. Biophys. Acta Mol. Cell Res. 1868, 118876. 33007331 10.1016/j.bbamcr.2020.118876 PMC7769147

[17] Kedersha N., Cho M.R., Li W., Yacono P.W., Chen S., Gilks N., Golan D.E., and Anderson P. (2000). Dynamic shuttling of TIA-1 accompanies the recruitment of mRNA to mammalian stress granules. J. Cell Biol. 151, 1257–1268. 11121440 10.1083/jcb.151.6.1257 PMC2190599

[18] Matheny T., Rao B.S., and Parker R. (2019). Transcriptome-Wide Comparison of Stress Granules and P-Bodies Reveals that Translation Plays a Major Role in RNA Partitioning. Mol. Cell. Biol. 39. 10.1128/MCB.00313-19. PMC6879202 31591142

[19] Matheny T., Van Treeck B., Huynh T.N., and Parker R. (2021). RNA partitioning into stress granules is based on the summation of multiple interactions. RNA 27, 174–189. 33199441 10.1261/rna.078204.120 PMC7812873

[20] Namkoong S., Ho A., Woo Y.M., Kwak H., and Lee J.H. (2018). Systematic Characterization of Stress-Induced RNA Granulation. Mol. Cell 70, 175–187.e8. 29576526 10.1016/j.molcel.2018.02.025 PMC6359928

[21] Guillén-Boixet J., Kopach A., Holehouse A.S., Wittmann S., Jahnel M., Schlüßler R., Kim K., Trussina I.R.E.A., Wang J., Mateju D., (2020). RNA-Induced Conformational Switching and Clustering of G3BP Drive Stress Granule Assembly by Condensation. Cell 181, 346–361.e17. 32302572 10.1016/j.cell.2020.03.049 PMC7181197

[22] Yang P., Mathieu C., Kolaitis R.-M., Zhang P., Messing J., Yurtsever U., Yang Z., Wu J., Li Y., Pan Q., (2020). G3BP1 Is a Tunable Switch that Triggers Phase Separation to Assemble Stress Granules. Cell 181, 325–345.e28. 32302571 10.1016/j.cell.2020.03.046 PMC7448383

[23] Moon S.L., Morisaki T., Khong A., Lyon K., Parker R., and Stasevich T.J. (2019). Multicolour single-molecule tracking of mRNA interactions with RNP granules. Nat. Cell Biol. 21, 162–168. 30664789 10.1038/s41556-018-0263-4 PMC6375083

[24] Preiss T., Baron-Benhamou J., Ansorge W., and Hentze M.W. (2003). Homodirectional changes in transcriptome composition and mRNA translation induced by rapamycin and heat shock. Nat. Struct. Biol. 10, 1039–1047. 14608375 10.1038/nsb1015

[25] Wallace E.W.J., Kear-Scott J.L., Pilipenko E.V., Schwartz M.H., Laskowski P.R., Rojek A.E., Katanski C.D., Riback J.A., Dion M.F., Franks A.M., (2015). Reversible, Specific, Active Aggregates of Endogenous Proteins Assemble upon Heat Stress. Cell 162, 1286–1298. 26359986 10.1016/j.cell.2015.08.041 PMC4567705

[26] Kroschwald S., Munder M.C., Maharana S., Franzmann T.M., Richter D., Ruer M., Hyman A.A., and Alberti S. (2018). Different Material States of Pub1 Condensates Define Distinct Modes of Stress Adaptation and Recovery. Cell Rep. 23, 3327–3339. 29898402 10.1016/j.celrep.2018.05.041

[27] Iserman C., Altamirano C.D., Jegers C., Friedrich U., Zarin T., Fritsch A.W., Mittasch M., Domingues A., Hersemann L., Jahnel M., (2020). Condensation of Ded1p Promotes a Translational Switch from Housekeeping to Stress Protein Production. Cell 0. 10.1016/j.cell.2020.04.009. PMC7237889 32359423

[28] Franzmann T.M., Jahnel M., Pozniakovsky A., Mahamid J., Holehouse A.S., Nüske E., Richter D., Baumeister W., Grill S.W., Pappu R.V., (2018). Phase separation of a yeast prion protein promotes cellular fitness. Science 359, eaao5654. 29301985 10.1126/science.aao5654

[29] Grousl T., Ivanov P., Malcova I., Pompach P., Frydlova I., Slaba R., Senohrabkova L., Novakova L., and Hasek J. (2013). Heat shock-induced accumulation of translation elongation and termination factors precedes assembly of stress granules in S. cerevisiae. PLoS One 8, e57083. 23451152 10.1371/journal.pone.0057083 PMC3581570

[30] Glauninger H., Wong Hickernell C.J., Bard J.A.M., and Drummond D.A. (2022). Stressful steps: Progress and challenges in understanding stress-induced mRNA condensation and accumulation in stress granules. Molecular Cell 82, 2544–2556. 35662398 10.1016/j.molcel.2022.05.014 PMC9308734

[31] Wallace E.W.J., Kear-Scott J.L., Pilipenko E.V., Schwartz M.H., Laskowski P.R., Rojek A.E., Katanski C.D., Riback J.A., Dion M.F., Franks A.M., (2015). Reversible, Specific, Active Aggregates of Endogenous Proteins Assemble upon Heat Stress. Cell 162, 1286–1298. 26359986 10.1016/j.cell.2015.08.041 PMC4567705

[32] Keyport Kik S., Christopher D., Glauninger H., Hickernell C.W., Bard J.A.M., Ford M., Sosnick T.R., and Allan Drummond D. (2023). An adaptive biomolecular condensation response is conserved across environmentally divergent species. bioRxiv, 2023.07.28.551061. 10.1101/2023.07.28.551061. PMC11009240 38605014

[33] Blobel G. (1971). Isolation of a 5S RNA-protein complex from mammalian ribosomes. Proc. Natl. Acad. Sci. U. S. A. 68, 1881–1885. 5001943 10.1073/pnas.68.8.1881 PMC389313

[34] Nolan R.D., and Arnstein H.R. (1969). The dissociation of rabbit reticulocyte ribosomes into subparticles active in protein synthesis. Eur. J. Biochem. 10, 96–101. 5345988 10.1111/j.1432-1033.1969.tb00660.x

[35] Riback J.A., Katanski C.D., Kear-Scott J.L., Pilipenko E.V., Rojek A.E., Sosnick T.R., and Drummond D.A. (2017). Stress-Triggered Phase Separation Is an Adaptive, Evolutionarily Tuned Response. Cell 168, 1028–1040.e19. 28283059 10.1016/j.cell.2017.02.027 PMC5401687

[36] Khong A., Matheny T., Jain S., Mitchell S.F., Wheeler J.R., and Parker R. (2017). The Stress Granule Transcriptome Reveals Principles of mRNA Accumulation in Stress Granules. Mol. Cell 68, 808–820.e5. 29129640 10.1016/j.molcel.2017.10.015 PMC5728175

[37] Van Treeck B., Protter D.S.W., Matheny T., Khong A., Link C.D., and Parker R. (2018). RNA self-assembly contributes to stress granule formation and defining the stress granule transcriptome. Proc. Natl. Acad. Sci. U. S. A., 201800038. 10.1073/pnas.1800038115 PMC5856561 29483269

[38] Campos-Melo D., Hawley Z.C.E., Droppelmann C.A., and Strong M.J. (2021). The Integral Role of RNA in Stress Granule Formation and Function. Front Cell Dev Biol 9, 621779. 34095105 10.3389/fcell.2021.621779 PMC8173143

[39] Moon S.L., Morisaki T., Stasevich T.J., and Parker R. (2020). Coupling of translation quality control and mRNA targeting to stress granules. J. Cell Biol. 219. 10.1083/jcb.202004120. PMC7401812 32520986

[40] Pincus D., Anandhakumar J., Thiru P., Guertin M.J., Erkine A.M., and Gross D.S. (2018). Genetic and epigenetic determinants establish a continuum of Hsf1 occupancy and activity across the yeast genome. Mol. Biol. Cell 29, 3168–3182. 30332327 10.1091/mbc.E18-06-0353 PMC6340206

[41] Solís E.J., Pandey J.P., Zheng X., Jin D.X., Gupta P.B., Airoldi E.M., Pincus D., and Denic V. (2016). Defining the Essential Function of Yeast Hsf1 Reveals a Compact Transcriptional Program for Maintaining Eukaryotic Proteostasis. Mol. Cell 63, 60–71. 27320198 10.1016/j.molcel.2016.05.014 PMC4938784

[42] Femino A.M., Fay F.S., Fogarty K., and Singer R.H. (1998). Visualization of single RNA transcripts in situ. Science 280, 585–590. 9554849 10.1126/science.280.5363.585

[43] Buchan J.R., Yoon J.H., and Parker R. (2011). Stress-specific composition, assembly and kinetics of stress granules in Saccharomyces cerevisiae. J. Cell Sci. 124, 228–239. 21172806 10.1242/jcs.078444 PMC3010191

[44] Jain S., Wheeler J.R., Walters R.W., Agrawal A., Barsic A., and Parker R. (2016). ATPase-Modulated Stress Granules Contain a Diverse Proteome and Substructure. Cell 164, 487–498. 26777405 10.1016/j.cell.2015.12.038 PMC4733397

[45] Eiermann N., Stoecklin G., and Jovanovic B. (2022). Mitochondrial Inhibition by Sodium Azide Induces Assembly of eIF2α Phosphorylation-Independent Stress Granules in Mammalian Cells. Int. J. Mol. Sci. 23, 5600. 35628412 10.3390/ijms23105600 PMC9142010

[46] Kato K., Yamamoto Y., and Izawa S. (2011). Severe ethanol stress induces assembly of stress granules in Saccharomyces cerevisiae. Yeast 28, 339–347. 21341306 10.1002/yea.1842

[47] Arava Y., Wang Y., Storey J.D., Liu C.L., Brown P.O., and Herschlag D. (2003). Genome-wide analysis of mRNA translation profiles in Saccharomyces cerevisiae. Proc. Natl. Acad. Sci. U. S. A. 100, 3889–3894. 12660367 10.1073/pnas.0635171100 PMC153018

[48] Mendoza-Ochoa G.I., Barrass J.D., Terlouw B.R., Maudlin I.E., de Lucas S., Sani E., Aslanzadeh V., Reid J.A.E., and Beggs J.D. (2019). A fast and tuneable auxin-inducible degron for depletion of target proteins in budding yeast. Yeast 36, 75–81. 30375036 10.1002/yea.3362 PMC6587778

[49] Yesbolatova A., Saito Y., Kitamoto N., Makino-Itou H., Ajima R., Nakano R., Nakaoka H., Fukui K., Gamo K., Tominari Y., (2020). The auxin-inducible degron 2 technology provides sharp degradation control in yeast, mammalian cells, and mice. Nat. Commun. 11, 5701. 33177522 10.1038/s41467-020-19532-z PMC7659001

[50] Weinberg D.E., Shah P., Eichhorn S.W., Hussmann J.A., Plotkin J.B., and Bartel D.P. (2016). Improved Ribosome-Footprint and mRNA Measurements Provide Insights into Dynamics and Regulation of Yeast Translation. Cell Rep. 14, 1787–1799. 26876183 10.1016/j.celrep.2016.01.043 PMC4767672

[51] Weenink T., van der Hilst J., McKiernan R.M., and Ellis T. (2018). Design of RNA hairpin modules that predictably tune translation in yeast. Synth. Biol. 3, ysy019. 10.1093/synbio/ysy019 PMC7445769 32995525

[52] Sanders D.W., Kedersha N., Lee D.S.W., Strom A.R., Drake V., Riback J.A., Bracha D., Eeftens J.M., Iwanicki A., Wang A., (2020). Competing Protein-RNA Interaction Networks Control Multiphase Intracellular Organization. Cell 181, 306–324.e28. 32302570 10.1016/j.cell.2020.03.050 PMC7816278

[53] Hinnebusch A.G. (2005). Translational regulation of GCN4 and the general amino acid control of yeast. Annu. Rev. Microbiol. 59, 407–450. 16153175 10.1146/annurev.micro.59.031805.133833

[54] Cox J.S., and Walter P. (1996). A novel mechanism for regulating activity of a transcription factor that controls the unfolded protein response. Cell 87, 391–404. 8898193 10.1016/s0092-8674(00)81360-4

[55] Mueller P.P., and Hinnebusch A.G. (1986). Multiple upstream AUG codons mediate translational control of GCN4. Cell 45, 201–207. 3516411 10.1016/0092-8674(86)90384-3

[56] Hata T., Ishiwata-Kimata Y., and Kimata Y. (2022). Induction of the Unfolded Protein Response at High Temperature in Saccharomyces cerevisiae. Int. J. Mol. Sci. 23. 10.3390/ijms23031669. PMC8836091 35163590

[57] Kimata Y., Ishiwata-Kimata Y., Yamada S., and Kohno K. (2006). Yeast unfolded protein response pathway regulates expression of genes for anti-oxidative stress and for cell surface proteins. Genes Cells 11, 59–69. 16371132 10.1111/j.1365-2443.2005.00921.x

[58] Mühlhofer M., Berchtold E., Stratil C.G., Csaba G., Kunold E., Bach N.C., Sieber S.A., Haslbeck M., Zimmer R., and Buchner J. (2019). The Heat Shock Response in Yeast Maintains Protein Homeostasis by Chaperoning and Replenishing Proteins. Cell Rep. 29, 4593–4607.e8. 31875563 10.1016/j.celrep.2019.11.109

[59] Mazroui R., Sukarieh R., Bordeleau M.-E., Kaufman R.J., Northcote P., Tanaka J., Gallouzi I., and Pelletier J. (2006). Inhibition of ribosome recruitment induces stress granule formation independently of eukaryotic initiation factor 2alpha phosphorylation. Mol. Biol. Cell 17, 4212–4219. 16870703 10.1091/mbc.E06-04-0318 PMC1635342

[60] Tauber D., Tauber G., Khong A., Van Treeck B., Pelletier J., and Parker R. (2020). Modulation of RNA Condensation by the DEAD-Box Protein eIF4A. Cell 180, 411–426.e16. 31928844 10.1016/j.cell.2019.12.031 PMC7194247

[61] Zhou C., Slaughter B.D., Unruh J.R., Guo F., Yu Z., Mickey K., Narkar A., Ross R.T., McClain M., and Li R. (2014). Organelle-Based Aggregation and Retention of Damaged Proteins in Asymmetrically Dividing Cells. Cell 159, 530–542. 25417105 10.1016/j.cell.2014.09.026 PMC6726438

[62] Mazroui R., Huot M.-E., Tremblay S., Filion C., Labelle Y., and Khandjian E.W. (2002). Trapping of messenger RNA by Fragile X Mental Retardation protein into cytoplasmic granules induces translation repression. Hum. Mol. Genet. 11, 3007–3017. 12417522 10.1093/hmg/11.24.3007

[63] Jain A., and Vale R.D. (2017). RNA phase transitions in repeat expansion disorders. Nature 546, 243–247. 28562589 10.1038/nature22386 PMC5555642

[64] Ripin N., and Parker R. (2022). Are stress granules the RNA analogs of misfolded protein aggregates? RNA 28, 67–75. 34670846 10.1261/rna.079000.121 PMC8675284

[65] Fedorovskiy A.G., Burakov A.V., Terenin I.M., Bykov D.A., Lashkevich K.A., Popenko V.I., Makarova N.E., Sorokin I.I., Sukhinina A.P., Prassolov V.S., (2023). A solitary stalled 80S ribosome prevents mRNA recruitment to stress granules. Biochemistry (Mosc.) 88, 1786–1799. 38105199 10.1134/S000629792311010X

[66] Putnam A., Thomas L., and Seydoux G. (2023). RNA granules: functional compartments or incidental condensates? Genes Dev. 37, 354–376. 37137715 10.1101/gad.350518.123 PMC10270194

[67] Chan L.Y., Mugler C.F., Heinrich S., Vallotton P., and Weis K. (2018). Non-invasive measurement of mRNA decay reveals translation initiation as the major determinant of mRNA stability. Elife 7. 10.7554/eLife.32536. PMC6152797 30192227

[68] Panas M.D., Ivanov P., and Anderson P. (2016). Mechanistic insights into mammalian stress granule dynamics. J. Cell Biol. 215, 313–323. 27821493 10.1083/jcb.201609081 PMC5100297

[69] Vilela C., Velasco C., Ptushkina M., and McCarthy J.E. (2000). The eukaryotic mRNA decapping protein Dcp1 interacts physically and functionally with the eIF4F translation initiation complex. EMBO J. 19, 4372–4382. 10944120 10.1093/emboj/19.16.4372 PMC302023

[70] Wang J., Johnson A.G., Lapointe C.P., Choi J., Prabhakar A., Chen D.-H., Petrov A.N., and Puglisi J.D. (2019). eIF5B gates the transition from translation initiation to elongation. Nature 573, 605–608. 31534220 10.1038/s41586-019-1561-0 PMC6763361

[71] Yoon J.H., Choi E.J., and Parker R. (2010). Dcp2 phosphorylation by Ste20 modulates stress granule assembly and mRNA decay in Saccharomyces cerevisiae. J. Cell Biol. 189, 813–827. 20513766 10.1083/jcb.200912019 PMC2878948

[72] Hubstenberger A., Courel M., Bénard M., Souquere S., Ernoult-Lange M., Chouaib R., Yi Z., Morlot J.-B., Munier A., Fradet M., (2017). P-Body Purification Reveals the Condensation of Repressed mRNA Regulons. Mol. Cell 68, 144–157.e5. 28965817 10.1016/j.molcel.2017.09.003

[73] Rao B.S., and Parker R. (2017). Numerous interactions act redundantly to assemble a tunable size of P bodies in *Saccharomyces cerevisiae*. Proceedings of the National Academy of Sciences 114, E9569–E9578. 10.1073/pnas.1712396114 PMC5692575 29078371

[74] Zedan M., Schuerch A.P., Heinrich S., Garcia P.G., Khawaja S., and Weis K. (2024). Newly synthesized mRNA selectively escapes translational repression following acute stress. bioRxiv, 2024.04.14.589419. 10.1101/2024.04.14.589419.

[75] Garre E., Romero-Santacreu L., De Clercq N., Blasco-Angulo N., Sunnerhagen P., and Alepuz P. (2012). Yeast mRNA cap-binding protein Cbc1/Sto1 is necessary for the rapid reprogramming of translation after hyperosmotic shock. Mol. Biol. Cell 23, 137–150. 22072789 10.1091/mbc.E11-05-0419 PMC3248893

[76] Escalante L.E., and Gasch A.P. (2021). The role of stress-activated RNA-protein granules in surviving adversity. RNA. 10.1261/rna.078738.121. PMC8208049 33931500

[77] Cherkasov V., Grousl T., Theer P., Vainshtein Y., Gläßer C., Mongis C., Kramer G., Stoecklin G., Knop M., Mogk A., (2015). Systemic control of protein synthesis through sequestration of translation and ribosome biogenesis factors during severe heat stress. FEBS Lett. 10.1016/j.febslet.2015.10.010. 26484595

[78] Walters R.W., Muhlrad D., Garcia J., and Parker R. (2015). Differential effects of Ydj1 and Sis1 on Hsp70-mediated clearance of stress granules in Saccharomyces cerevisiae. RNA 21, 1660–1671. 26199455 10.1261/rna.053116.115 PMC4536325

[79] Yoo H., Bard J.A.M., Pilipenko E.V., and Drummond D.A. (2022). Chaperones directly and efficiently disperse stress-triggered biomolecular condensates. Mol. Cell 82, 741–755.e11. 35148816 10.1016/j.molcel.2022.01.005 PMC8857057

[80] Zheng X., Krakowiak J., Patel N., Beyzavi A., Ezike J., Khalil A.S., and Pincus D. (2016). Dynamic control of Hsf1 during heat shock by a chaperone switch and phosphorylation. Elife 5. 10.7554/eLife.18638. PMC5127643 27831465

[81] Krakowiak J., Zheng X., Patel N., Feder Z.A., Anandhakumar J., Valerius K., Gross D.S., Khalil A.S., and Pincus D. (2018). Hsf1 and Hsp70 constitute a two-component feedback loop that regulates the yeast heat shock response. Elife 7. 10.7554/eLife.31668. PMC5809143 29393852

[82] Gietz R.D., and Woods R.A. (2002). Transformation of yeast by lithium acetate/single-stranded carrier DNA/polyethylene glycol method. Methods Enzymol. 350, 87–96. 12073338 10.1016/s0076-6879(02)50957-5

[83] Cherry J.M., Hong E.L., Amundsen C., Balakrishnan R., Binkley G., Chan E.T., Christie K.R., Costanzo M.C., Dwight S.S., Engel S.R., (2012). Saccharomyces Genome Database: the genomics resource of budding yeast. Nucleic Acids Res. 40, D700–D705. 22110037 10.1093/nar/gkr1029 PMC3245034

[84] Rutherford K.M., Lera-Ramírez M., and Wood V. (2024). PomBase: a Global Core Biodata Resource-growth, collaboration, and sustainability. Genetics. 10.1093/genetics/iyae007. PMC11075564 38376816

[85] Dobin A., Davis C.A., Schlesinger F., Drenkow J., Zaleski C., Jha S., Batut P., Chaisson M., and Gingeras T.R. (2013). STAR: ultrafast universal RNA-seq aligner. Bioinformatics 29, 15–21. 23104886 10.1093/bioinformatics/bts635 PMC3530905

[86] Bray N.L., Pimentel H., Melsted P., and Pachter L. (2016). Near-optimal probabilistic RNA-seq quantification. Nat. Biotechnol. 34, 525–527. 27043002 10.1038/nbt.3519

[87] Smith T., Heger A., and Sudbery I. (2017). UMI-tools: modeling sequencing errors in Unique Molecular Identifiers to improve quantification accuracy. Genome Res. 27, 491–499. 28100584 10.1101/gr.209601.116 PMC5340976

[88] Li H., Handsaker B., Wysoker A., Fennell T., Ruan J., Homer N., Marth G., Abecasis G., and Durbin R. (2009). The Sequence Alignment/Map format and SAMtools. Bioinformatics 25, 2078–2079. 19505943 10.1093/bioinformatics/btp352 PMC2723002

[89] Robert C.P., and Casella G. Monte Carlo Statistical Methods (Springer New York).

[90] Stan Development Team (2023). RStan: the R interface to Stan. Preprint.

[91] R Core Team (2022). R: A Language and Environment for Statistical Computing. Preprint at R Foundation for Statistical Computing.

[92] Pelechano V., Wei W., and Steinmetz L.M. (2013). Extensive transcriptional heterogeneity revealed by isoform profiling. Nature 497, 127–131. 23615609 10.1038/nature12121 PMC3705217

[93] Love M.I., Huber W., and Anders S. (2014). Moderated estimation of fold change and dispersion for RNA-seq data with DESeq2. Genome Biol. 15, 550. 25516281 10.1186/s13059-014-0550-8 PMC4302049

[94] Ristau J., Watt K., Oertlin C., and Larsson O. (2022). Polysome Fractionation for Transcriptome-Wide Studies of mRNA Translation. Methods Mol. Biol. 2418, 223–241. 35119669 10.1007/978-1-0716-1920-9_14

[95] Chothani S., Adami E., Ouyang J.F., Viswanathan S., Hubner N., Cook S.A., Schafer S., and Rackham O.J.L. (2019). deltaTE: Detection of Translationally Regulated Genes by Integrative Analysis of Ribo-seq and RNA-seq Data. Curr. Protoc. Mol. Biol. 129, e108. 31763789 10.1002/cpmb.108 PMC9285699

[96] Lorenz R., Bernhart S.H., Höner Zu Siederdissen C., Tafer H., Flamm C., Stadler P.F., and Hofacker I.L. (2011). ViennaRNA Package 2.0. Algorithms Mol. Biol. 6, 26. 22115189 10.1186/1748-7188-6-26 PMC3319429

[97] Azizoglu A., Brent R., and Rudolf F. (2021). A precisely adjustable, variation-suppressed eukaryotic transcriptional controller to enable genetic discovery. Elife 10. 10.7554/eLife.69549. PMC8421071 34342575

[98] Lee M.E., DeLoache W.C., Cervantes B., and Dueber J.E. (2015). A Highly Characterized Yeast Toolkit for Modular, Multipart Assembly. ACS Synth. Biol. 4, 975–986. 25871405 10.1021/sb500366v

[99] Masser A.E., Kandasamy G., Kaimal J.M., and Andréasson C. (2016). Luciferase NanoLuc as a reporter for gene expression and protein levels in Saccharomyces cerevisiae. Yeast 33, 191–200. 26860732 10.1002/yea.3155 PMC5069653

[100] Akhmetov A., Laurent J.M., Gollihar J., Gardner E.C., Garge R.K., Ellington A.D., Kachroo A.H., and Marcotte E.M. (2018). Single-step Precision Genome Editing in Yeast Using CRISPR-Cas9. Bio Protoc 8. 10.21769/BioProtoc.2765. PMC5951413 29770349

[101] Triandafillou C.G., Katanski C.D., Dinner A.R., and Drummond D.A. (2020). Transient intracellular acidification regulates the core transcriptional heat shock response. Elife 9. 10.7554/eLife.54880. PMC7449696 32762843

[102] Kushnirov V.V. (2000). Rapid and reliable protein extraction from yeast. Yeast 16, 857–860. 10861908 10.1002/1097-0061(20000630)16:9<857::AID-YEA561>3.0.CO;2-B

[103] Li W., Maekiniemi A., Sato H., Osman C., and Singer R.H. (2022). An improved imaging system that corrects MS2-induced RNA destabilization. Nat. Methods 19, 1558–1562. 36357695 10.1038/s41592-022-01658-1 PMC7613886

[104] Rahman S., and Zenklusen D. (2013). Single-molecule resolution fluorescent in situ hybridization (smFISH) in the yeast S. cerevisiae. Methods Mol. Biol. 1042, 33–46. 23979998 10.1007/978-1-62703-526-2_3

[105] Kopalle H. (2019). Visualization of membrane-less granules in yeast and mammalian cells using modified fluorescence in-situ hybridization.

[106] Imbert A., Ouyang W., Safieddine A., Coleno E., Zimmer C., Bertrand E., Walter T., and Mueller F. (2021). FISH-quant v2: a scalable and modular analysis tool for smFISH image analysis. bioRxiv, 2021.07.20.453024. 10.1101/2021.07.20.453024. PMC9074904 35347070

