# Supplementary material for: Transcriptome-wide mRNA condensation precedes stress granule formation and excludes stress-induced transcripts
Source: bioRxiv. 2024 May 2:2024.04.15.589678. Preprint. [Version 3] doi: 10.1101/2024.04.15.589678 (PMC11042329; doi:10.1101/2024.04.15.589678)
Supplement: Supplementary file 1 [file media-1.pdf]

Supplementary information accompanying “Transcriptome-wide mRNA condensation precedes stress granule formation and excludes stress-induced transcripts”

Glauninger, Bard, Wong Hickernell *et al*. 2024

Supplementary Text

A model of free mRNP sedimentation

Let L be the length of an mRNA under consideration. We seek the functional form of $\mathrm{pSup}(L)$, the proportion in the supernatant after centrifugation, which will depend on many factors. Several of these are experimental and we assume they do not change across samples or mRNAs, such as the spin speed ω, the sample height h, the spin time s, and the viscosity η . Terminal velocity for a particle with mass m and hydration radius r_0 is:

$$v_t(m, r_0) = \frac{m\omega^2 r}{6\pi\eta r_0} = \left(\frac{m}{r_0}\right)\left(\frac{\omega^2 r}{6\pi\eta}\right)$$

We assume that both mass m and hydration radius r_0 scale consistently with length L on average. In the case of mass, we assume proportional scaling: a constant (average) molecular weight per unit length deriving from nucleotides and bound proteins. In the case of hydration radius, our scaling assumption is consistent with standard approaches (Yoffe et al. 2008), for the radius of gyration R_g scales as L^χ with $\chi = 0.34$ for naked ssRNA due to secondary structure. The hydration radius r_0 will be proportional to R_g assuming a constant density. The scaling relationship will also depend on the geometry of the vessel.

Assuming a constant mass per nucleotide and protein binding per unit length, the mass of an mRNP scales linearly with L. Therefore, the leading term above will tend to scale with L:

$$\frac{m}{r_0} = (c_1 L)^\chi$$

with proportionality constant c_1 taking care of the specific conversion factors (e.g. the molecular weight per nucleotide) and with χ summarizing the scaling.

Then the proportion of the particle species remaining in the supernatant is the proportion of the tube which is more than $v_t s$ from the bottom. That is: $v_t s$ from the bottom. That is:

$$\mathrm{pSup}(m, r_0, h, s) = \begin{cases} 0 & v_t(m, r_0)s \geq h \\ 1 - \frac{v_t(m,r_0)s}{h} & 0 \leq v_t(m, r_0)s < h \end{cases}$$

We can then summarize the dependencies with two constants, as follows.

$$\begin{aligned} \mathrm{pSup}(L) &= 1 - \frac{v_t(m, r_0)s}{h} \\ &= 1 - \left(\frac{m}{r_0}\right)\left(\frac{\omega^2 r}{6\pi\eta}\right) \\ &= 1 - c_2(c_1 L)^{\chi} \\ &= 1 - \beta L^{\chi} \end{aligned}$$

That is, two parameters should be sufficient to describe the average behavior of free (unclustered) mRNAs of length L. A simple test of this model arises from a particular prediction: by rearranging, we see that

$$\log(1 - \mathrm{pSup}(L)) = \log\beta + \chi\log L$$

which predicts a linear relationship between $\log(1 - \mathrm{pSup}(L))$ and $\log L$. Indeed, our data show this relationship (Figure S1B).

A model of mRNP clustering

To model between-mRNP interactions and their effect on pSup, we consider two kinds of interactions, both assumed to be Poisson-distributed with rates which vary across conditions and potentially across transcripts.

The first interaction type is between nucleotides, including direct RNA-RNA interactions and interactions mediated by proteins but dependent on nucleotide content; these are expected to be length-dependent. Consequently, we model them by a per-nucleotide interaction rate ν, such that the probability that an mRNP has k nucleotide-dependent interactions is

$$\Pr(k) = (\nu L)^k e^{-\nu L}/k!.$$

The second interaction type is between molecules and is independent of sequence length, including interactions mediated by the 5' cap or the 3' end. We model these as a per-molecule interaction rate μ such that the probability that an mRNP has m such interactions is

$$\Pr(m) = \mu^m e^{-\mu}/m!.$$

These rates permit us to model the probability that an mRNP of a given length L engages in interactions with other mRNPs, which may have varying lengths/sizes and may themselves also engage in additional interactions, making prediction of sedimentation highly complex. To proceed analytically, we make a simplifying assumption: that under the conditions we study, most mRNP clusters are sufficiently large to sediment completely ($\mathrm{pSup}(\mathrm{cluster}) = 0$). Under this assumption, the observed proportion in the supernatant for an mRNA of length L is given by the free-mRNP pSup multiplied by the probability that the mRNP is free. The latter probability is equal to the probability of $n = 0$ per-nucleotide interactions and $m = 0$ per-molecule interactions. Together, we have

$$\mathrm{pSup}(L) = (1 - \beta L^{\chi})\, e^{-(\mu+\nu L)}.$$

The mean behavior of transcripts in any particular experiment, in this model, can be derived from only four fitted parameters β, χ, μ, ν and the lengths of all transcripts. Fits of this model are shown in Fig. 1G, where the no-stress sample is modeled with no clustering ($\mu = 0$, $\nu = 0$) and stress samples are modeled with clustering (solid lines) and also in comparison to curves where the per-molecule interaction rate is zero ($\mu = 0$; dashed lines).

Bounds on condensate sizes

How can we put bounds on the average size of condensates, given an observed value of pSup? Any pSup value can be realized in a range of ways, bounded by two extremes. At one extreme, the molecular population is homogenous (all molecules of a particular type are in condensates of the same size), and all clusters have the same probability of remaining in the supernatant, which is pSup. At the other extreme, the molecular population is heterogeneous, with each species of condensate having some proportion in the supernatant $pSup_i$ and proportion of the molecular population q_i. Order the indices *i* such that the lightest species (monomers) has *i=1* and all condensates are ordered by their size (and hence in the descending order of their pSup) up to *n*. First, if *n*=2, it is clear that the observed $pSup \geq pSup_2$, simply because the average of two numbers is great than or equal to the smaller of the two numbers. Second, if we define the average of the condensates' pSups as $pSup_{cond}$, we have

$$pSup_{cond} = \frac{1}{1-q_1}\sum_{i>1}^{n} q_i\, pSup_i$$

So that

$$pSup = q_1\, pSup_1 + (1 - q_1) pSup_{cond} \geq q_1\, pSup_{cond} + (1 - q_1) pSup_{cond} = pSup_{cond}$$

which establishes $pSup \geq pSup_{cond}$ such that in general, pSup is an upper bound on the average condensate size.

Alternatives to condensation

We considered the possibility changes in mRNA length during stress, either due to splicing or changes in polyadenylation, explain these solubility changes. Fewer than 5% of yeast genes contain introns, so splicing cannot account for the transcriptome-scale effects we observe. Polyadenylation yields poly(A) tails of only ~50 nucleotides on average (3% of the median transcript length) which, in high-expression genes, grow slightly shorter upon heat stress (Tudek et al. 2021), not longer as would be required to decrease pSup during stress.

What about an increase in mRNP mass due to changes in protein binding? While formally possible, this would not plausibly increase free mRNP sizes by more than tenfold as we observe during severe stress. We provisionally conclude that changes in the physical size of otherwise free mRNPs cannot account for apparent sedimentation changes.

What about stress-induced binding to a large sedimentable structure, such as the ER, which has been argued to be the site of substantial protein synthesis of cytosolic proteins (Reid and Nicchitta 2015)? While we cannot rule all possibilities out, our data suggest this is not the case. Most importantly, substantial transcriptome-wide mRNA clustering occurs while poly(A)+ mRNA appears diffuse and primarily cytosolic (42°C, Fig. 1B/C), inconsistent with localization to the ER or another structure. Once clusters become visually resolvable—i.e., stress granules form—these are well-known to be distinct structures.

Simulation of complex condensation

To assess whether assumed effects produce observed phenomena, we turned to a detailed simulation of condensation and sedimentation (available along with other analytical code; see “Data and code availability” section in the main manuscript). Direct simulation of interactions, consequent formation of condensates in a fully heterogeneous population, and sedimentation of these heterogeneous condensates according to sedimentation theory by the model above, yields the same trends as in Fig. 1G (Fig. S1D,E). Fig. S1E shows a qualitatively excellent reproduction of the major trends in the biological heat-shock data (Fig. 1D), using only one parameter which varies during stress: the rate of interaction per molecule, which can arise e.g. through interactions mediated by the 5’ or 3’ ends of transcripts (see main text). Importantly, the simulation does not invoke a key assumption used in the analytical model above, which is that all condensates sediment completely.

Supplementary Figures

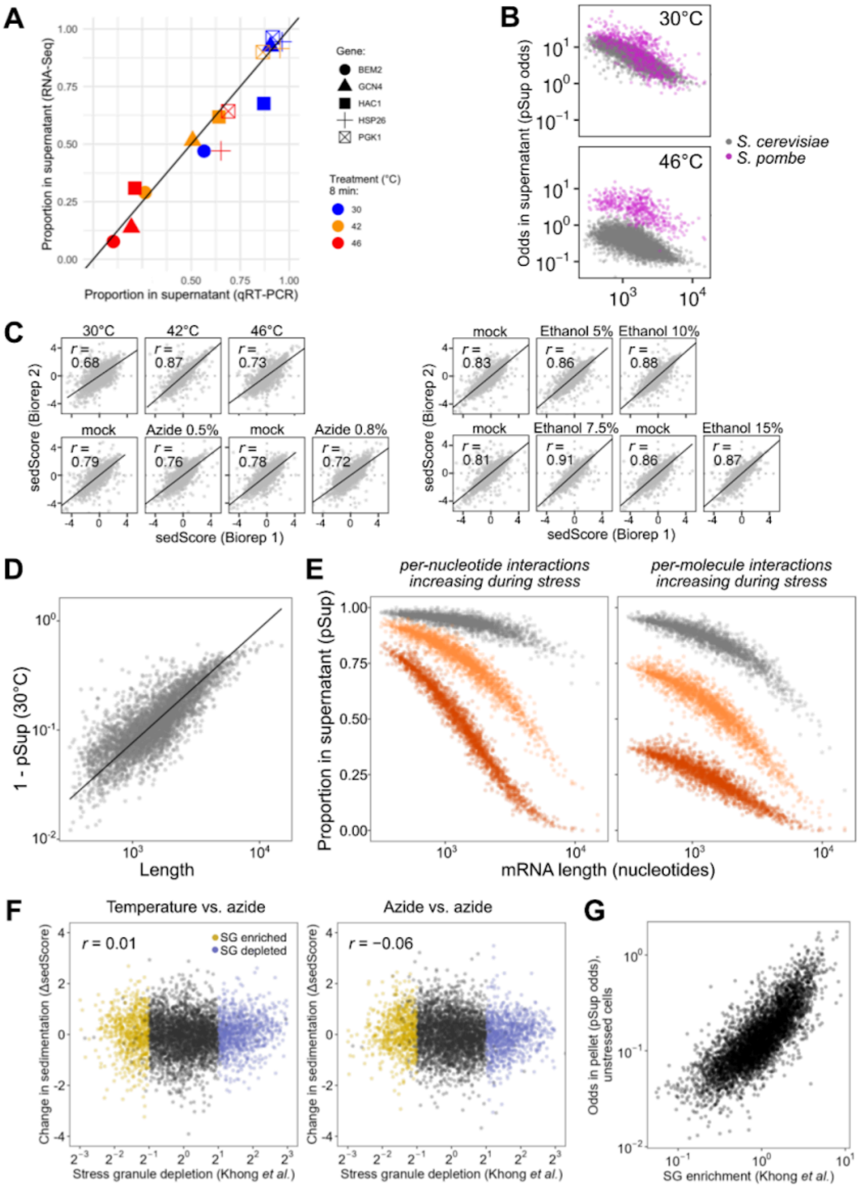

Figure S1: **Sed-seq captures previously unreported mRNA condensation largely driven by length-independent mechanisms.** (A) The measurements of mRNA proportion in supernatant (pSup) from RNA sequencing match pSups calculated using spike-in-normalized abundances quantified by RT-qPCR. (B) Purified mRNA (*S. pombe* total RNA) spiked into lysate from unstressed yeast cells recapitulates length-dependent sedimentation of free mRNA, and remains largely free even when spiked into lysate from severely stressed yeast cells. (C) Across stresses, length-normalized sedimentation (sedScore) of biological replicates correlate well *(r* = pearson correlation coefficient). (D) A simple model for the length-dependence of sedimentation for free mRNPs predicts a linear dependence between log(1-pSup) and log(length), which is observed (*r* = 0.81). (E) Simulations of mRNA clustering with only per-nucleotide (length-dependent) interactions permitted, at increasing rates of interaction, and simulations of mRNA clustering with only per-molecule (length-independent) interactions permitted, at increasing rates of interaction. (F) Negligible correlation (Pearson) is observed between reported depletion from azide-induced stress granules (Khong *et al.* 2017) and exclusion from stress-induced clusters (change in sedScore) after heat stress or azide stress. (G) Most of the variation in stress-granule depletion in a previous study can be explained by sedimentation of free mRNPs due to their length, and thus can be closely reproduced using only Sed-seq data from unstressed cells.

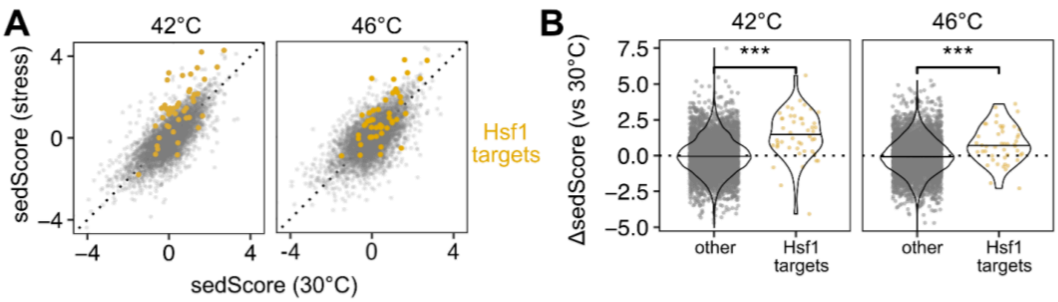

Figure S2: (A) sedScores at 30°C largely match sedScores during heat shock, but the stress-induced regulon exhibits increased sedScores during stress reflecting escape from global condensation. (B) Hsf1-regulated transcripts have higher sedScores at both 42°C and 46°C compared with the rest of the transcriptome. (Wilcoxon rank sum test: *** = $P < 0.001$)

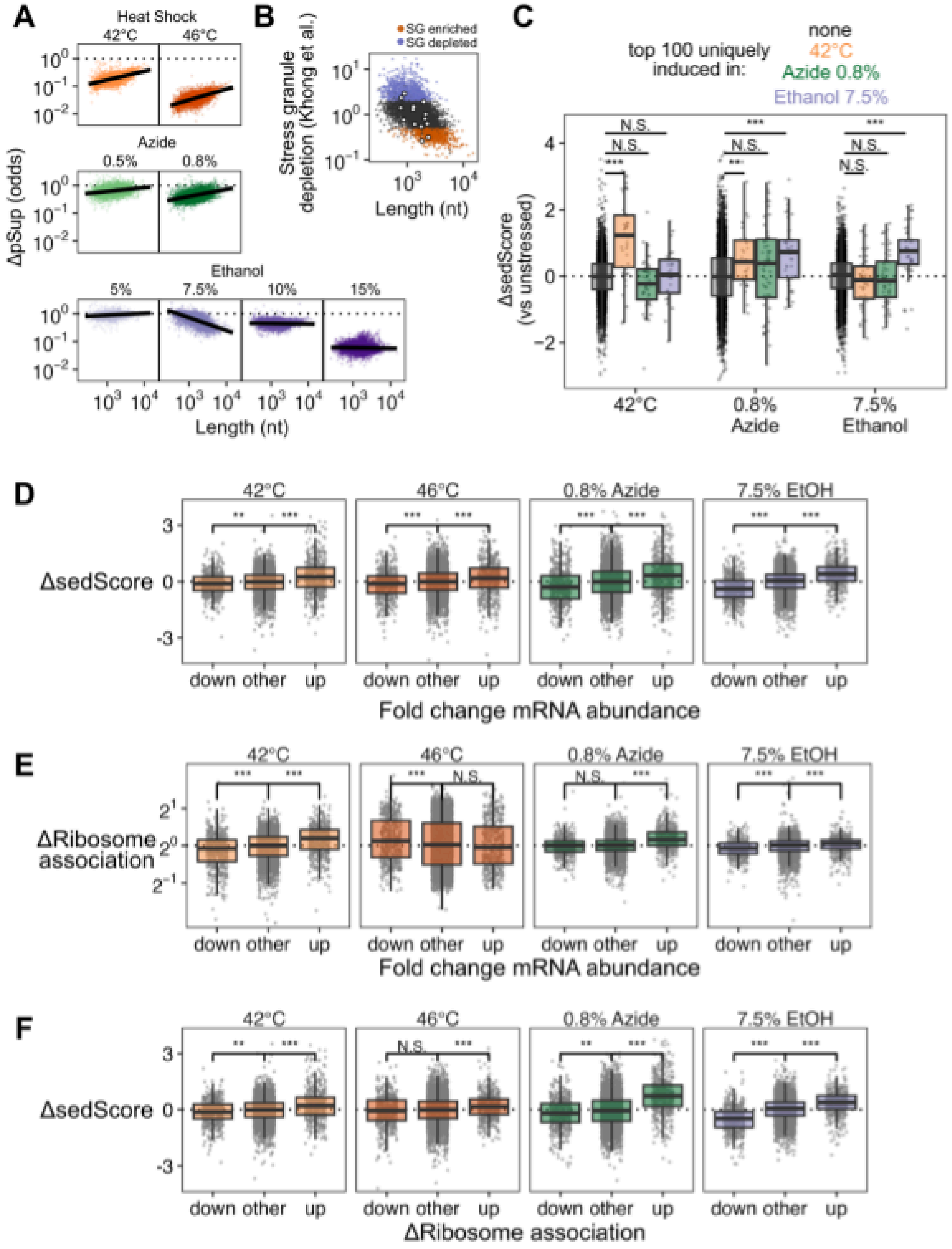

Figure S3: (A) Length dependence of the change in sedimentation compared across stresses. In general, shorter transcripts increase in sedimentation more than long ones. (B) Induced transcripts are not particularly depleted from stress granules based on data from Khong *et al.*, 2017. Points in white are the top 1% of induced transcripts during azide stress. (C) The change in sedimentation of the top 100 uniquely induced transcripts across three stresses are compared. During heat shock and ethanol stress, only those transcripts which are specifically induced escape condensation. (D) Comparison of ΔsedScore grouped by the 10% most induced and repressed transcripts during stress. (E) Comparison of the change in ribosome association grouped by the 10% most induced and repressed transcripts during stress. (F) Comparison of ΔsedScore grouped by the top and bottom 10% transcripts with changing ribosome association during stress. (Wilcoxon rank sum with Bonferroni correction: N.S. = $P \geq 0.05$, ** = $P < 0.01$, *** = $P < 0.001$)

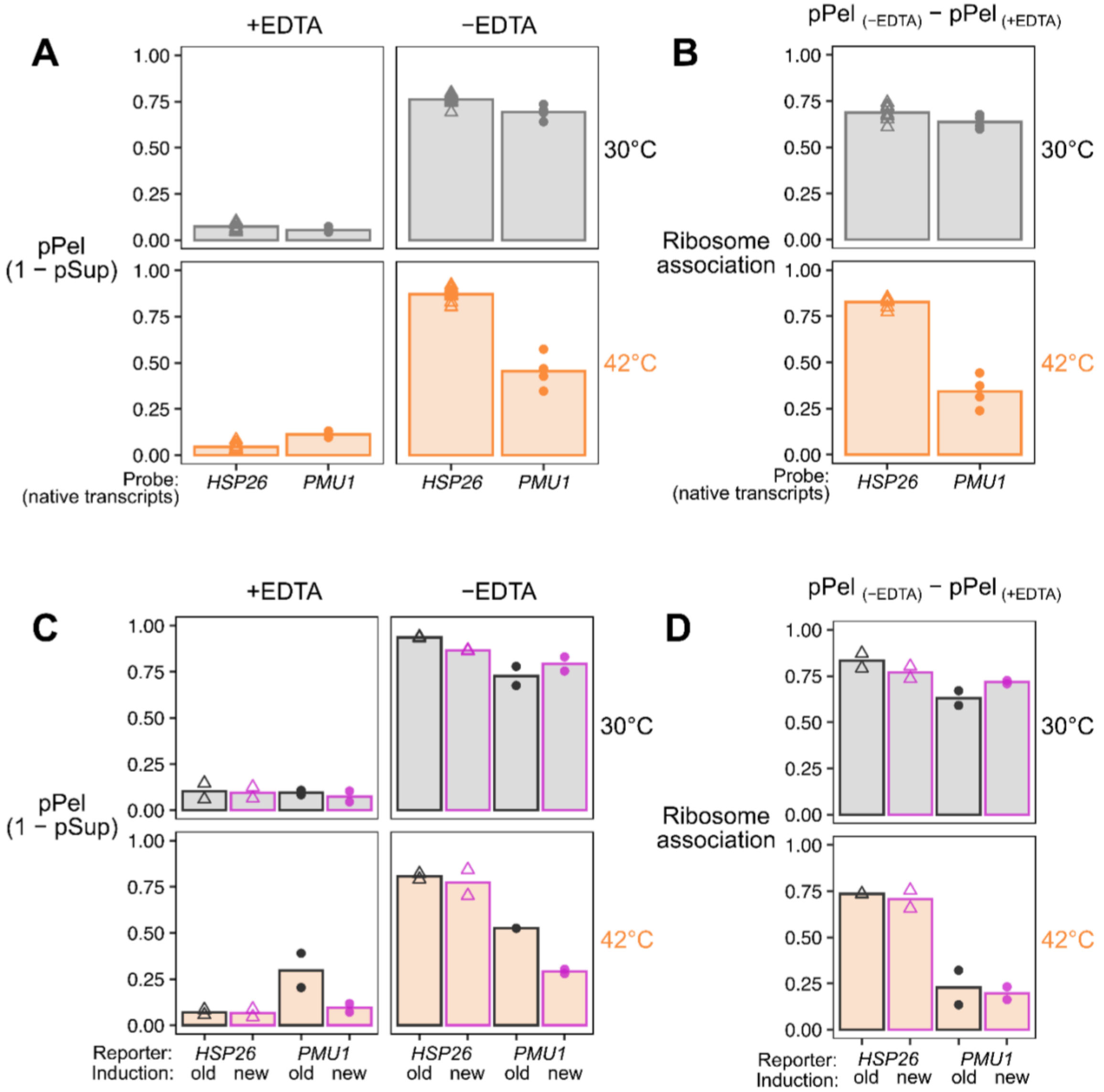

Figure S4: (A) Cellular lysate from stressed (20 minutes at 42°C) or unstressed yeast was centrifuged through a 1M sucrose cushion with and without EDTA. The proportion of mRNA transcripts in the pellet was quantified with qPCR, normalized to spike-in RNA. (B) In the absence of EDTA, transcripts can pellet either because they are bound to ribosomes or because they are condensed, however ribosomes fall off transcripts in the presence of EDTA. Thus to calculate the ribosome occupancy, the pPel (+EDTA) is subtracted from the pPel (−EDTA). (C,D) The same samples as in (A) and (B), but tracking the abundance of the *HSP26* and *PMU1* reporters induced before stress (old) or after stress (new).

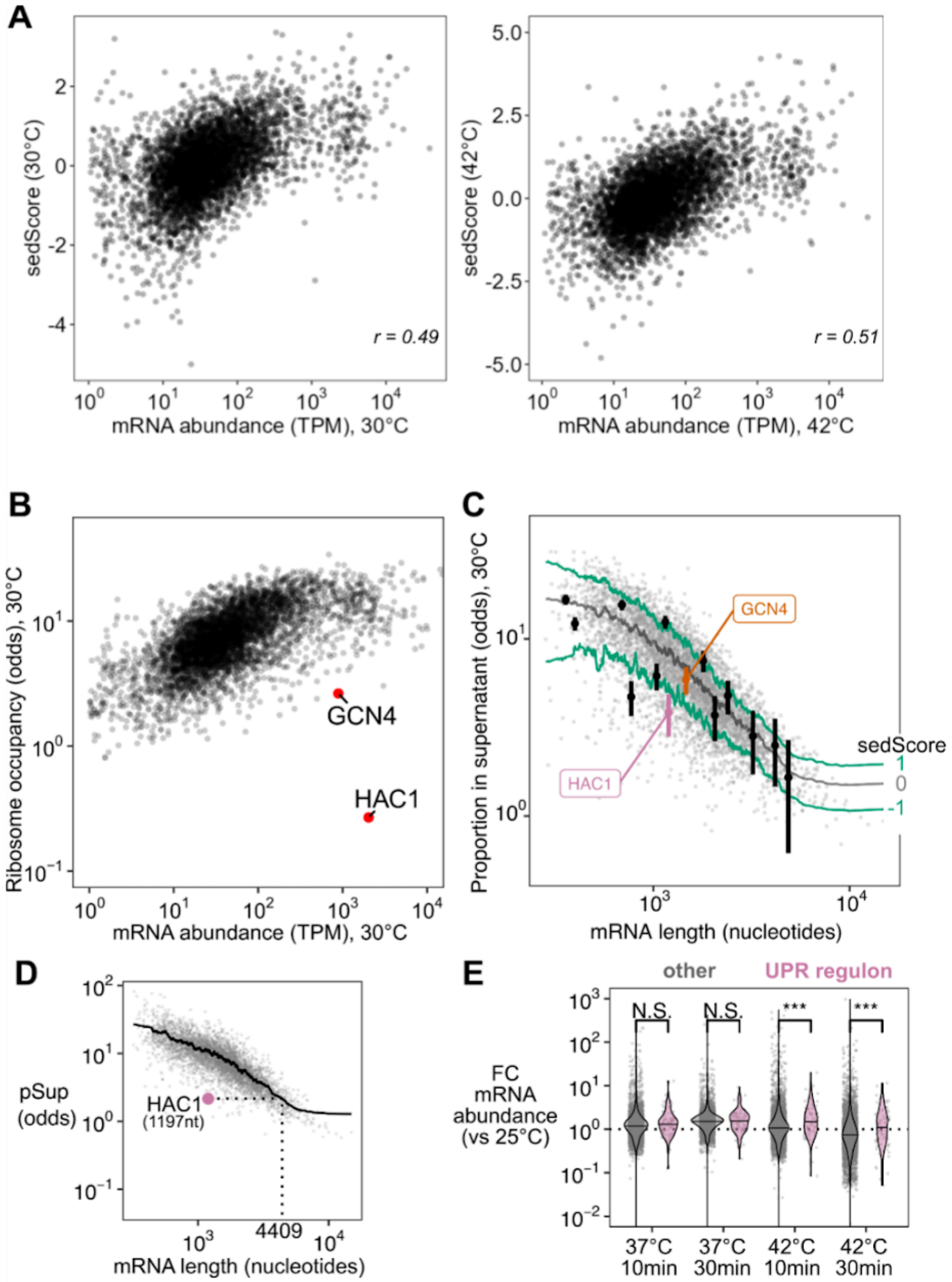

Figure S5: (A) SedScores in unstressed (30°C) and stressed (42°C) cells correlate with mRNA abundance. Higher-expressed transcripts show lower levels of relative condensation (higher sedScores). Correlations shown are Spearman r, $P < 10^{-6}$. (B) *HAC1* and *GCN4* transcripts stand out as being highly abundant, but poorly translated using ribosome occupancy data in unstressed cells reported in this manuscript. (C) *HAC1* has a consistently lower pSup (shown here in log-odds). Sedimentation data is from 7 different replicates of unstressed yeast with different hairpin reporters, as shown in Figure 5B. Each point represents the geometric mean of the transcript pSup across replicates. In addition to *HAC1* and *GCN4* transcripts, a random selection of genes are highlighted, with their standard error across replicates shown. Also shown is the pSup value corresponding to a sedScore of -1, 0 or 1 for each nucleotide length (see Methods). (D) *HAC1* mRNA sediments with a similar rate to transcripts that are ~4x its length. (E) Reanalysis of Muhlhofer et al. 2020 RNA-seq data confirms that the UPR is upregulated after a 42°C stress but not a 37°C stress.

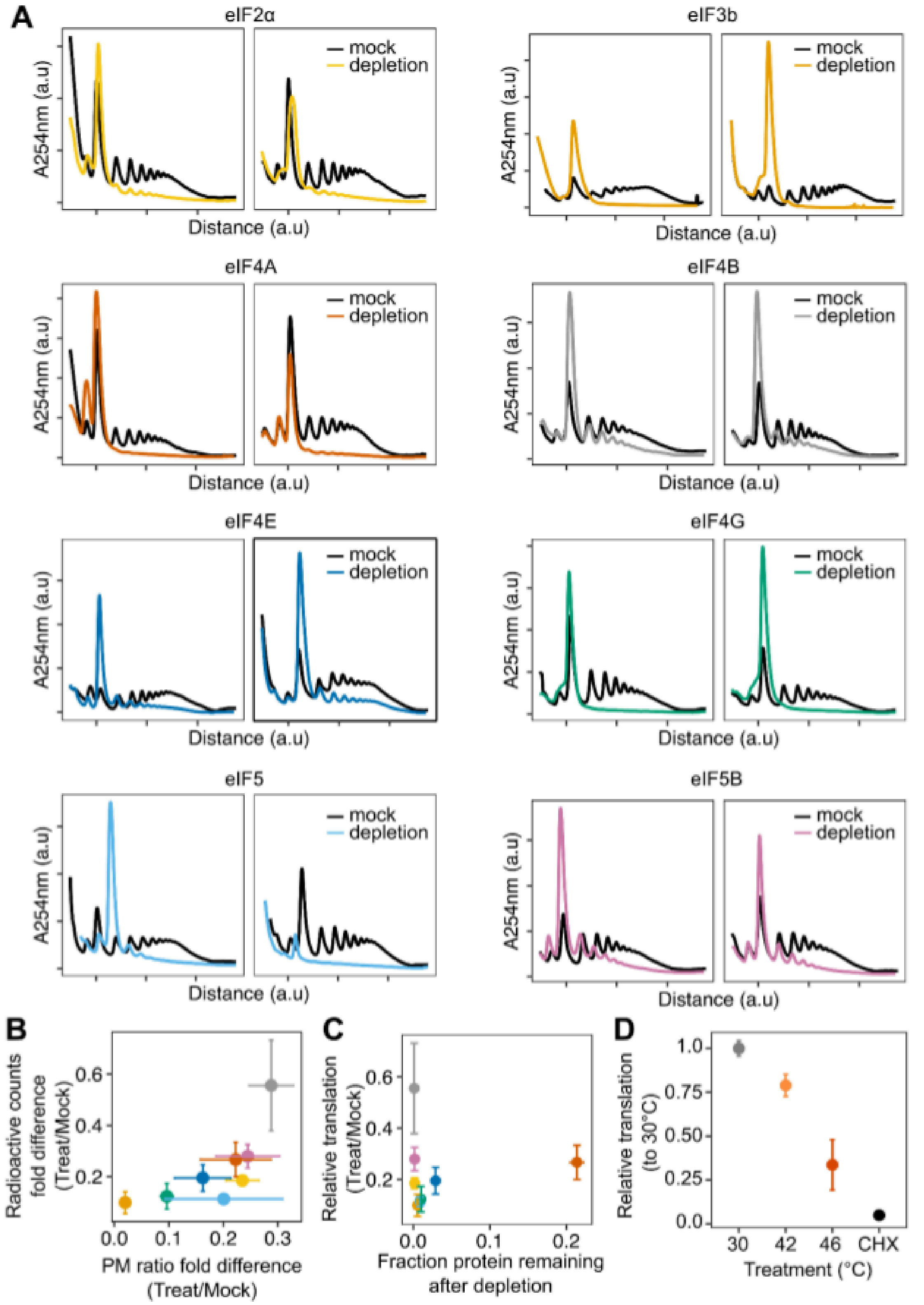

Figure S6: (A) Polysome profiles of AID depletion strains (B) Comparing the different measures of translation, radiolabeled amino acid incorporation and polysome profiling. (C) Comparing relative translation via radiolabelling vs. the amount of each protein remaining after the AID depletion. (D) Measures of relative translation via radiolabeled amino acid incorporation after 15 minutes of 30°C, 42°C, 46°C, or 100 μg/mL CHX.

Table S1: Yeast strains used in this study

Yeast strain	Description	Genotype	Source
BY4741	background strain S288C	MATa ura3Δ0 leu2Δ0 his3Δ1 met15Δ0	(Brachmann et al. 1998)
BY4742	background strain S288C	MATα ura3Δ0 leu2Δ0 his3Δ1 lys2Δ0	(Brachmann et al. 1998)
yJB001	Pab1-Halotag	MATa ura3Δ0 leu2Δ0 his3Δ1 met15Δ0 pab1::PAB1-HaloTag	This manuscript
yAER020	Pab1-Clover	MATα ura3Δ0 leu2Δ0 his3Δ1 lys2Δ0 pab1::PAB1-Clover pTEF-KanMX	(Wallace et al. 2015)
yJB42 (eIF3b)	PRT1-AID*-3xflag+pZTRL	MATα ura3Δ0 leu2::pZ4EV-OsTIR1_Z4EV-ATF_LEU2 his3Δ1 lys2Δ0 PRT1::PRT1-AID*-3xflag	This manuscript
yHG55 (eIF4b)	AID*-TIF3-3xflag+pZTRL	MATα ura3Δ0 leu2::pZ4EV-OsTIR1_Z4EV-ATF_LEU2 his3Δ1 lys2Δ0 TIF3::TIF3-AID*-3xflag	This manuscript
yHG38 (eIF4E)	CDC33-AID*-3xflag+pZTRL	MATα ura3Δ0 leu2::pZ4EV-OsTIR1_Z4EV-ATF_LEU2 his3Δ1 lys2Δ0 CDC33::CDC33-AID*-3xflag	This manuscript
yHG72 (eIF4A)	TIF1-AID*-3xflag+pZTRL	MATα ura3Δ0 leu2::pZ4EV-OsTIR1_Z4EV-ATF_LEU2 his3Δ1 lys2Δ0 TIF1::TIF1-AID*-3xflag tif2Δ::KANMX	This manuscript
yJB143 (eIF2α)	SUI2-AID-3xFlag + pJB773	MATα ura3Δ0 leu2::pZ4EV-OsTIR1F74G_Z4EV-ATF_LEU2 his3Δ1 lys2Δ0 SUI2::SUI2-AID*-3xflag	This manuscript
yJB147 (eIf5)	TIF5-AID-3xFlag + pJB773	MATα ura3Δ0 leu2::pZ4EV-OsTIR1F74G_Z4EV-ATF_LEU2 his3Δ1 lys2Δ0 TIF5::TIF5-AID*-3xflag	This manuscript
yJB148 (eIF5B)	AID*-FUN12-3xFlag + pJB773	MATα ura3Δ0 leu2::pZ4EV-OsTIR1F74G_Z4EV-ATF_LEU2 his3Δ1 lys2Δ0 FUN12::AID-FUN12*-3xflag	This manuscript
yJB262 (eIF4G)	TIF4631-AID*-3xFLAG+pJB773	MATα ura3Δ0 leu2::pZ4EV-OsTIR1F74G_Z4EV-ATF_LEU2 his3Δ1 lys2Δ0	This manuscript

		TIF4631::TIF4631-AID*-3xFLAG tif4632Δ::KANMX	
Pbp1-GFP	Pbp1-GFP	MATa his3Δ1 leu2Δ0 met15Δ0 ura3Δ0 pbp1:: Pbp1-GFP	(Huh et al. 2003)
yJB265	yJB42+Pab1-Clover	MATɑ ura3Δ0 leu2::pZ4EV-OsTIR1_Z4EV-ATF_LEU2 his3Δ1 lys2Δ0 PRT1::PRT1-AID*-3xflag pab1::PAB1-Clover	This manuscript
yHG005	Weakest hairpin	MATa ura3Δ0 his3Δ1 met15Δ0 leu2::LEU2_5′weakest hairpin-cds2xClover-3′TPI1_5′TPI1-cdsmCherry-3′TPI1	This manuscript
yHG006	Weak hairpin	MATa ura3Δ0 his3Δ1 met15Δ0 leu2::LEU2_5′weak hairpin-cds2xClover-3′TPI1_5′TPI1-cdsmCherry-3′TPI1	This manuscript
yHG007	Medium harpin	MATa ura3Δ0 his3Δ1 met15Δ0 leu2::LEU2_5′medium hairpin-cds2xClover-3′TPI1_5′TPI1-cdsmCherry-3′TPI1	This manuscript
yHG008	Strongest hairpin	MATa ura3Δ0 his3Δ1 met15Δ0 leu2::LEU2_5′strongest hairpin-cds2xClover-3′TPI1_5′TPI1-cdsmCherry-3′TPI1	This manuscript
yHG010	No hairpin	MATa ura3Δ0 his3Δ1 met15Δ0 leu2::LEU2_5′no hairpin-cds2xClover-3′TPI1_5′TPI1-cdsmCherry-3′TPI1	This manuscript
yHG026	GCN4 reporter	MATa ura3Δ0 his3Δ1 met15Δ0 leu2::LEU2_5′GCN4-cds2xClover-3′TPI1_5′TPI1-cdsmCherry-3′TPI1	This manuscript
yHG027	GCN4 5xmut	MATa ura3Δ0 his3Δ1 met15Δ0 leu2::LEU2_5′GCN4(5xmut)-cds2xClover-3′TPI1_5′TPI1-cdsmCherry-3′TPI1	This manuscript
yJB236	Tet inducible PMU1 reporter	MATɑ ura3Δ0 leu2::pZ4EV-OsTIR1_Z4EV-ATF_LEU2 his3::TetR,TetR-Tup1,HIS3MX lys2Δ0 SUI2::SUI2-AID*-3xflag ho::pTETO-5′PMU1-cdsPMU1-nanoluc-3′PMU1,TPI-firefly,hygR	This manuscript
yJB240	Tet inducible HSP26	MATɑ ura3Δ0	This manuscript

	reporter	leu2::pZ4EV-OsTIR1_Z4EV-ATF_LEU2 his3::TetR,TetR-Tup1,HIS3MX lys2Δ0 SUI2::SUI2-AID*-3xflag ho::pTETO-5′HSP26-cdsTPI1-nanoluc-3′HSP26,TPI-firefly,hygR	

Table S2: Plasmids used in this study

**Plasmid**	**Description**	**Source**
pV1382	Cas9 editing	(Vyas et al. 2018)
pZTRL	Inducible OsTIR1	(Mendoza-Ochoa et al. 2019)
pJB773	OsTIR1F74G leu2 int leu	This manuscript
FRP2371	TETO system	(Azizoglu, Brent, and Rudolf 2021)
pyHG005	Weakest hairpin	This manuscript
pyHG006	Weak hairpin	This manuscript
pyHG007	Medium harpin	This manuscript
pyHG008	Strongest hairpin	This manuscript
pyHG010	No hairpin	This manuscript
pyHG026	GCN4 reporter	This manuscript
pyHG027	GCN4 5xmut	This manuscript
pJB801	Tet inducible PMU1 reporter	This manuscript
pJB805	Tet inducible HSP26 reporter	This manuscript

Table S3: smFISH probes used in this study

**Gene**	**Number**	**Sequence**	**Dye**
SSA4	1	cacatgaataggttgtacct	Quasar 670
SSA4	2	tcaaccctatcgtttgcaaa	Quasar 670
SSA4	3	acataagaaggcgtcgttct	Quasar 670
SSA4	4	agcctttctgtgtcagtaaa	Quasar 670

SSA4	5	tattatgtgggttcatcgca	Quasar 670
SSA4	6	ggatcatcgaatttacgtcc	Quasar 670
SSA4	7	taatgcttagcatcgttcgt	Quasar 670
SSA4	8	cccttgtcaatcactttgaa	Quasar 670
SSA4	9	tctttgtctcgcctttatat	Quasar 670
SSA4	10	ataggctggaaccgttacta	Quasar 670
SSA4	11	gattgtaccggcatcttttg	Quasar 670
SSA4	12	tacgaagaacgttcaagccc	Quasar 670
SSA4	13	gcagctgtaggttcattaat	Quasar 670
SSA4	14	ttctgcgatttcttgtccag	Quasar 670
SSA4	15	aaagatcaagacgttgtgct	Quasar 670
SSA4	16	ccttcatctatggatagcag	Quasar 670
SSA4	17	agaaagttaaccagcctact	Quasar 670
SSA4	18	ttttcttttgaactcctcgg	Quasar 670
SSA4	19	ggttagttgttagatccttt	Quasar 670
SSA4	20	tccttaacctccttagggac	Quasar 670
SSA4	21	tttctatagatgtctgagca	Quasar 670
SSA4	22	aattcttcaaatcttgccct	Quasar 670
SSA4	23	atcagccaaaactttttcca	Quasar 670
SSA4	24	accagtttttgtacttttgg	Quasar 670
SSA4	25	cctcatcagggttaatcgaa	Quasar 670
SSA4	26	ctgtacggcagcaccataag	Quasar 670
SSA4	27	actggtcacccgttaagatg	Quasar 670
SSA4	28	cagtaaatcttgggtcgtcg	Quasar 670
SSA4	29	ataatggtgcaacatccagc	Quasar 670
SSA4	30	ttttttgttgggatagtcga	Quasar 670
SSA4	31	gtaggtggaaaacacttccg	Quasar 670
SSA4	32	tttcaccctcaaaaacttgt	Quasar 670
SSA4	33	caactcaaatttacccagta	Quasar 670
SSA4	34	tcaacggcagatacgttcag	Quasar 670
SSA4	35	tcttcccttatcgttagtaa	Quasar 670
SSA4	36	aacttttctgcctcagcaac	Quasar 670
SSA4	37	gattcttagcttgaacacgt	Quasar 670
SSA4	38	gtaaacgcgtacgattctag	Quasar 670
SSA4	39	ttcgctcacagaatttttca	Quasar 670

SSA4	40	ccaccttctccttgaagtta	Quasar 670
SSA4	41	aatttcctggcatcctcttc	Quasar 670
SSA4	42	atttatagcatcttgggcgg	Quasar 670
SSA4	43	ccgcttgcgaagcatctaac	Quasar 670
SSA4	44	ataatggggtttgcaacacc	Quasar 670
SSA4	45	ctgcagctccgtaaaattta	Quasar 670
SSB1	1	taccgatagcaccttggaaa	Quasar 670
SSB1	2	tttcttgggttcaaagcagc	Quasar 670
SSB1	3	gtcgtcgaatcttctaccaa	Quasar 670
SSB1	4	gtcgataaccttgaaaggcc	Quasar 670
SSB1	5	tcttggtttcttccaagtat	Quasar 670
SSB1	6	agcggaaatttcttgtgggg	Quasar 670
SSB1	7	accaatcttagcttcagcaa	Quasar 670
SSB1	8	ggttcgttgatgatacgcaa	Quasar 670
SSB1	9	cacctagaccgtaagcaata	Quasar 670
SSB1	10	aacatgtctttccttttcgg	Quasar 670
SSB1	11	accagcaatgtgcaacaagg	Quasar 670
SSB1	12	gtgttaccggaagtagattt	Quasar 670
SSB1	13	agtgttccaacaagttggtg	Quasar 670
SSB1	14	ttcttcttgaattcagcctt	Quasar 670
SSB1	15	atcgtcggagatgtccaaac	Quasar 670
SSB1	16	aaggttctcttagctctttc	Quasar 670
SSB1	17	acggtagtttgagtgacaga	Quasar 670
SSB1	18	ggattcgaaatcttcaccgt	Quasar 670
SSB1	19	aacaatgcggcgttcaagtc	Quasar 670
SSB1	20	cttagagatcttagcatcct	Quasar 670
SSB1	21	ccaaccaagacaacttcgtc	Quasar 670
SSB1	22	tccaattgcttaccgtcaaa	Quasar 670
SSB1	23	acaacaagtccttggtttcg	Quasar 670
SSB1	24	gttcttctcttgatggttgg	Quasar 670
SSB1	25	actgggaattgaacggtggt	Quasar 670
SSB1	26	tgggatgttcttcaagtcga	Quasar 670
SSB1	27	atagcttccaagactggttc	Quasar 670

SSB1	28	accgttagcatcaacttcga	Quasar 670
SSB1	29	cgacggcagtaaccttcaag	Quasar 670
SSB1	30	gaagacttaccggtagactt	Quasar 670
SSB1	31	agacaagactgggtcagtga	Quasar 670
SSB1	32	gacttggaacctctcttcaa	Quasar 670
SSB1	33	tcgatttgcaaagcagccaa	Quasar 670
SSB1	34	aacgagaagacatggccttg	Quasar 670
HSP104	1	taggtaagggactgatccat	Quasar 670
HSP104	2	caggttgctgttgaggaatt	Quasar 670
HSP104	3	cccaaagcataacttggagt	Quasar 670
HSP104	4	ttgaatcttagcagcgtctt	Quasar 670
HSP104	5	gcgctataaatgagtccttc	Quasar 670
HSP104	6	tggcctcaatatctacttga	Quasar 670
HSP104	7	accacgaagttcaagagctt	Quasar 670
HSP104	8	ccacgagagtcaattctagt	Quasar 670
HSP104	9	tccaaaggtgtgttcgtatc	Quasar 670
HSP104	10	cctgctcagtcatatcaatg	Quasar 670
HSP104	11	agggtcaagtttaccttgac	Quasar 670
HSP104	12	tcttatttcttcttcacggc	Quasar 670
HSP104	13	acctggctcaccaattaaac	Quasar 670
HSP104	14	ttagcgccttgtaagatagt	Quasar 670
HSP104	15	aatgcggccaaatctagact	Quasar 670
HSP104	16	cttcaagatgttagcagcgt	Quasar 670
HSP104	17	tcaattggcctctggacaaa	Quasar 670
HSP104	18	cttctttcaaaggcaccatc	Quasar 670
HSP104	19	cactgtttgtctcacacttg	Quasar 670
HSP104	20	gttgcagacctctcaatatg	Quasar 670
HSP104	21	actaaggcgctatccagaat	Quasar 670
HSP104	22	aacgcttggctaattgagca	Quasar 670
HSP104	23	ctggcaatcttctatatggc	Quasar 670
HSP104	24	tcttctggcttagaatctct	Quasar 670
HSP104	25	catcttcatctctctctaga	Quasar 670
HSP104	26	ctatctttagtggtggagtc	Quasar 670

HSP104	27	tgcaatgaagcttccttctg	Quasar 670
HSP104	28	gttgtcttagaggttccaat	Quasar 670
HSP104	29	tttgatatctgggatggcga	Quasar 670
HSP104	30	accacattttggatcatgga	Quasar 670
HSP104	31	atcttgcagctgtttcagaa	Quasar 670
HSP104	32	cccacgacttcagatgataa	Quasar 670
HSP104	33	acggcattggaaacagcttt	Quasar 670
HSP104	34	ccttggattagctaaacctg	Quasar 670
HSP104	35	tagccaattcagttttaccg	Quasar 670
HSP104	36	aatcgaccctgatcatcatg	Quasar 670
HSP104	37	acttagagaccgcatacttc	Quasar 670
HSP104	38	ttcatcgtacccgacataac	Quasar 670
HSP104	39	cggagtatggtttgtattgc	Quasar 670
HSP104	40	gtaattctaccgtcatccaa	Quasar 670
HSP104	41	ttggaacagtcgatcgtctt	Quasar 670
HSP104	42	aaatgttgcctaacagcacc	Quasar 670
HSP104	43	tttgctcgaatctctcttca	Quasar 670
HSP104	44	ggcctcttgagttaaattca	Quasar 670
HSP104	45	cccatatcatcggaataacc	Quasar 670
HSP104	46	tctttaagatccttagtgcc	Quasar 670
HSP104	47	cgagatttacccttcttcaa	Quasar 670
HSP104	48	tagtagcttcgtgatttggt	Quasar 670
ADD66	1	gtatgttcctttgggttttg	Quasar 670
ADD66	2	tactaatggcaacaccaggc	Quasar 670
ADD66	3	attgtggtatattccctacg	Quasar 670
ADD66	4	ttcagcagccagtcaatact	Quasar 670
ADD66	5	ttcccattcatttgcttgtg	Quasar 670
ADD66	6	tcgaatctaacgcctccaaa	Quasar 670
ADD66	7	cccacaaattccactaggta	Quasar 670
ADD66	8	atcctctggtctgtctaatg	Quasar 670
ADD66	9	tctttatatagcgagtcgct	Quasar 670
ADD66	10	caaagcgctgctatacttca	Quasar 670
ADD66	11	cctcgcttcttattgtagaa	Quasar 670

ADD66	12	ttcgttgctgtatggcaaaa	Quasar 670
ADD66	13	tagttaacggacacgagtgg	Quasar 670
ADD66	14	cggcaagatgatctctacga	Quasar 670
ADD66	15	ccgatatattatacttgctc	Quasar 670
ADD66	16	agagagtcccaaatgcatat	Quasar 670
ADD66	17	attttcatcctccattgcat	Quasar 670
ADD66	18	cctgtggacgtacaatcaca	Quasar 670
ADD66	19	aactctccgagcgaatatac	Quasar 670
ADD66	20	ctcagcttcatcgtcgaaat	Quasar 670
ADD66	21	tcgtttagatggaggttcga	Quasar 670
ADD66	22	gttattgaccatcgactctt	Quasar 670
ADD66	23	tcttggaaactggtaggagt	Quasar 670
ADD66	24	tcggctgatcaacggatatc	Quasar 670
ADD66	25	cgacgcattcagtatttgga	Quasar 670
ADD66	26	taatgctgcggagggcttta	Quasar 670
ADD66	27	attggccaaacaactacagt	Quasar 670
ADD66	28	gaatctaatgagttgtcccc	Quasar 670
ADD66	29	ggcgcgtttttgataacttt	Quasar 670
ADD66	30	ggcctgacaaactttacaat	Quasar 670
ADD66	31	gtatgcaccttgccatgata	Quasar 670
ADD66	32	aatttatctcttgcatccgc	Quasar 670
ADD66	33	tacttctcggtttcaattgt	Quasar 670
ADD66	34	gtggctatatatgcacttgt	Quasar 670
oligodT	1	tttttttttttttttttt	Quasar 570

Table S4: Sequencing information:

Lysate_sample	Temperature	Biorep	Strain	Treatment Group	Treatment	Experiment	Sequencing Group (see Methods)	spike-in
L115	30C	BR1	BY4741	PolySeq_0.8% Azide	Azide_0.8%_pH6.8	PolySeq	C	pombe
L117	30C	BR2	BY4741	PolySeq_0.8% Azide	Azide_0.8%_pH6.8	PolySeq	C	pombe

L114	30C	BR1	BY4741	PolySeq_0.8% Azide	mock	PolySeq	C	pombe
L116	30C	BR2	BY4741	PolySeq_0.8% Azide	mock	PolySeq	C	pombe
L51	30C	BR1	BY4741	PolySeq_10min	none	PolySeq	B	pombe
L54	30C	BR2	BY4741	PolySeq_10min	none	PolySeq	B	pombe
L52	42C	BR1	BY4741	PolySeq_10min	none	PolySeq	B	pombe
L55	42C	BR2	BY4741	PolySeq_10min	none	PolySeq	B	pombe
L53	46C	BR1	BY4741	PolySeq_10min	none	PolySeq	B	pombe
L56	46C	BR2	BY4741	PolySeq_10min	none	PolySeq	B	pombe
L123	30C	BR1	BY4741	PolySeq_7.5% EtOH	EtOH_7.5 %	PolySeq	C	pombe
L125	30C	BR2	BY4741	PolySeq_7.5% EtOH	EtOH_7.5 %	PolySeq	C	pombe
L122	30C	BR1	BY4741	PolySeq_7.5% EtOH	mock	PolySeq	C	pombe
L124	30C	BR2	BY4741	PolySeq_7.5% EtOH	mock	PolySeq	C	pombe
L82	30C	BR1	BY4741	Azide_0.5%_pH6.8	Azide_0.5%_pH6.8	SedSeq	B	nanoluc
L84	30C	BR2	BY4741	Azide_0.5%_pH6.8	Azide_0.5%_pH6.8	SedSeq	B	nanoluc
L81	30C	BR1	BY4741	Azide_0.5%_pH6.8	mock	SedSeq	B	nanoluc
L83	30C	BR2	BY4741	Azide_0.5%_pH6.8	mock	SedSeq	B	nanoluc
L107	30C	BR1	BY4741	azide3	Azide_0.8%_pH6.8	SedSeq	C	nanoluc
L109	30C	BR2	BY4741	azide3	Azide_0.8%_pH6.8	SedSeq	C	nanoluc
L106	30C	BR1	BY4741	azide3	mock	SedSeq	C	nanoluc
L108	30C	BR2	BY4741	azide3	mock	SedSeq	C	nanoluc
L33	30C	BR1	BY4741	CHX	CHX	SedSeq	B	nanoluc
L38	30C	BR2	BY4741	CHX	CHX	SedSeq	B	nanoluc
L31	30C	BR1	BY4741	CHX	mock	SedSeq	B	nanoluc
L35	30C	BR2	BY4741	CHX	mock	SedSeq	B	nanoluc
L39	42C	BR2	BY4741	CHX	CHX	SedSeq	B	nanoluc
L36	42C	BR2	BY4741	CHX	mock	SedSeq	B	nanoluc

L34	46C	BR1	BY4741	CHX	CHX	SedSeq	B	nanoluc
L40	46C	BR2	BY4741	CHX	CHX	SedSeq	B	nanoluc
L32	46C	BR1	BY4741	CHX	mock	SedSeq	B	nanoluc
L37	46C	BR2	BY4741	CHX	mock	SedSeq	B	nanoluc
L72	30C	BR1	yJB042	eIF3b_deplete	eIF3b_deplete	SedSeq	B	nanoluc
L78	30C	BR2	yJB042	eIF3b_deplete	eIF3b_deplete	SedSeq	B	nanoluc
L69	30C	BR1	yJB042	eIF3b_deplete	mock	SedSeq	B	nanoluc
L75	30C	BR2	yJB042	eIF3b_deplete	mock	SedSeq	B	nanoluc
L73	42C	BR1	yJB042	eIF3b_deplete	eIF3b_deplete	SedSeq	B	nanoluc
L79	42C	BR2	yJB042	eIF3b_deplete	eIF3b_deplete	SedSeq	B	nanoluc
L70	42C	BR1	yJB042	eIF3b_deplete	mock	SedSeq	B	nanoluc
L76	42C	BR2	yJB042	eIF3b_deplete	mock	SedSeq	B	nanoluc
L74	46C	BR1	yJB042	eIF3b_deplete	eIF3b_deplete	SedSeq	B	nanoluc
L80	46C	BR2	yJB042	eIF3b_deplete	eIF3b_deplete	SedSeq	B	nanoluc
L71	46C	BR1	yJB042	eIF3b_deplete	mock	SedSeq	B	nanoluc
L77	46C	BR2	yJB042	eIF3b_deplete	mock	SedSeq	B	nanoluc
L60	30C	BR1	yHG038	eIF4E_deplete	eIF4E_deplete	SedSeq	B	nanoluc
L66	30C	BR2	yHG038	eIF4E_deplete	eIF4E_deplete	SedSeq	B	nanoluc
L57	30C	BR1	yHG038	eIF4E_deplete	mock	SedSeq	B	nanoluc
L63	30C	BR2	yHG038	eIF4E_deplete	mock	SedSeq	B	nanoluc
L61	42C	BR1	yHG038	eIF4E_deplete	eIF4E_deplete	SedSeq	B	nanoluc
L67	42C	BR2	yHG038	eIF4E_deplete	eIF4E_deplete	SedSeq	B	nanoluc
L58	42C	BR1	yHG038	eIF4E_deplete	mock	SedSeq	B	nanoluc
L64	42C	BR2	yHG038	eIF4E_deplete	mock	SedSeq	B	nanoluc
L62	46C	BR1	yHG038	eIF4E_deplete	eIF4E_deplete	SedSeq	B	nanoluc
L68	46C	BR2	yHG038	eIF4E_deplete	eIF4E_deplete	SedSeq	B	nanoluc

L59	46C	BR1	yHG038	eIF4E_deplete	mock	SedSeq	B	nanoluc
L65	46C	BR2	yHG038	eIF4E_deplete	mock	SedSeq	B	nanoluc
L7	30C	BR1	BY4741	EW_TSPP	none	SedSeq	A	none
L11	30C	BR2	BY4741	EW_TSPP	none	SedSeq	A	none
L8	42C	BR1	BY4741	EW_TSPP	none	SedSeq	A	none
L12	42C	BR2	BY4741	EW_TSPP	none	SedSeq	A	none
L9	46C	BR1	BY4741	EW_TSPP	none	SedSeq	A	none
L13	46C	BR2	BY4741	EW_TSPP	none	SedSeq	A	none
L89	30C	BR1	yHG010	hairpinReporters	none	SedSeq	C	nanoluc
L90	30C	BR1	yHG005	hairpinReporters	none	SedSeq	C	nanoluc
L91	30C	BR1	yHG006	hairpinReporters	none	SedSeq	C	nanoluc
L92	30C	BR1	yHG007	hairpinReporters	none	SedSeq	C	nanoluc
L93	30C	BR1	yHG008	hairpinReporters	none	SedSeq	C	nanoluc
L94	30C	BR1	yHG026	hairpinReporters	none	SedSeq	C	nanoluc
L95	30C	BR1	yHG027	hairpinReporters	none	SedSeq	C	nanoluc
L43	30C	BR1	BY4741	HyperOsmotic_EtOH	EtOH_15%	SedSeq	B	nanoluc
L46	30C	BR2	BY4741	HyperOsmotic_EtOH	EtOH_15%	SedSeq	B	nanoluc
L41	30C	BR1	BY4741	HyperOsmotic_EtOH	mock	SedSeq	B	nanoluc
L44	30C	BR2	BY4741	HyperOsmotic_EtOH	mock	SedSeq	B	nanoluc
L98	30C	BR1	BY4741	milderEtOH1	EtOH_10%	SedSeq	C	nanoluc
L101	30C	BR2	BY4741	milderEtOH1	EtOH_10%	SedSeq	C	nanoluc
L97	30C	BR1	BY4741	milderEtOH1	EtOH_5%	SedSeq	C	nanoluc
L100	30C	BR2	BY4741	milderEtOH1	EtOH_5%	SedSeq	C	nanoluc
L96	30C	BR1	BY4741	milderEtOH1	mock	SedSeq	C	nanoluc
L99	30C	BR2	BY4741	milderEtOH1	mock	SedSeq	C	nanoluc

L103	30C	BR1	BY4741	milderEtOH2	EtOH_7.5%	SedSeq	C	nanoluc
L105	30C	BR2	BY4741	milderEtOH2	EtOH_7.5%	SedSeq	C	nanoluc
L102	30C	BR1	BY4741	milderEtOH2	mock	SedSeq	C	nanoluc
L104	30C	BR2	BY4741	milderEtOH2	mock	SedSeq	C	nanoluc
L110	30C	BR1	BY4741	spombe_spikeIn	none	SedSeq	C	nanoluc+pombe
L112	30C	BR2	BY4741	spombe_spikeIn	none	SedSeq	C	nanoluc+pombe
L111	46C	BR1	BY4741	spombe_spikeIn	none	SedSeq	C	nanoluc+pombe
L113	46C	BR2	BY4741	spombe_spikeIn	none	SedSeq	C	nanoluc+pombe
L49	30C	BR1	BY4741	uprInduction	DTT_10mM	SedSeq	B	nanoluc
L50	30C	BR2	BY4741	uprInduction	DTT_10mM	SedSeq	B	nanoluc
L47	30C	BR1	BY4741	uprInduction	mock	SedSeq	B	nanoluc
L48	30C	BR2	BY4741	uprInduction	mock	SedSeq	B	nanoluc

Azizoglu, Asli, Roger Brent, and Fabian Rudolf. 2021. “A Precisely Adjustable, Variation-Suppressed Eukaryotic Transcriptional Controller to Enable Genetic Discovery.” *eLife* 10 (August). https://doi.org/10.7554/eLife.69549.

Brachmann, C. B., A. Davies, G. J. Cost, E. Caputo, J. Li, P. Hieter, and J. D. Boeke. 1998. “Designer Deletion Strains Derived from Saccharomyces Cerevisiae S288C: A Useful Set of Strains and Plasmids for PCR-Mediated Gene Disruption and Other Applications.” *Yeast* 14 (2): 115–32.

Huh, Won-Ki, James V. Falvo, Luke C. Gerke, Adam S. Carroll, Russell W. Howson, Jonathan S. Weissman, and Erin K. O’Shea. 2003. “Global Analysis of Protein Localization in Budding Yeast.” *Nature* 425 (6959): 686–91.

Mendoza-Ochoa, Gonzalo I., J. David Barrass, Barbara R. Terlouw, Isabella E. Maudlin, Susana de Lucas, Emanuela Sani, Vahid Aslanzadeh, Jane A. E. Reid, and Jean D. Beggs. 2019. “A Fast and Tuneable Auxin-Inducible Degron for Depletion of Target Proteins in Budding Yeast.” *Yeast* 36 (1): 75–81.

Reid, David W., and Christopher V. Nicchitta. 2015. “Diversity and Selectivity in mRNA Translation on the Endoplasmic Reticulum.” *Nature Reviews. Molecular Cell Biology* 16 (4): 221–31.

Tudek, Agnieszka, Paweł S. Krawczyk, Seweryn Mroczek, Rafał Tomecki, Matti Turtola, Katarzyna Matylla-Kulińska, Torben Heick Jensen, and Andrzej Dziembowski. 2021. “Global View on the Metabolism of RNA poly(A) Tails in Yeast Saccharomyces Cerevisiae.” *Nature Communications* 12 (1): 4951.

Vyas, Valmik K., G. Guy Bushkin, Douglas A. Bernstein, Matthew A. Getz, Magdalena Sewastianik, M. Inmaculada Barrasa, David P. Bartel, and Gerald R. Fink. 2018. “New

CRISPR Mutagenesis Strategies Reveal Variation in Repair Mechanisms among Fungi." *mSphere* 3 (2). https://doi.org/10.1128/mSphere.00154-18.
Wallace, Edward W. J., Jamie L. Kear-Scott, Evgeny V. Pilipenko, Michael H. Schwartz, Pawel R. Laskowski, Alexandra E. Rojek, Christopher D. Katanski, et al. 2015. "Reversible, Specific, Active Aggregates of Endogenous Proteins Assemble upon Heat Stress." *Cell* 162 (6): 1286–98.
Yoffe, Aron M., Peter Prinsen, Ajaykumar Gopal, Charles M. Knobler, William M. Gelbart, and Avinoam Ben-Shaul. 2008. "Predicting the Sizes of Large RNA Molecules." *Proceedings of the National Academy of Sciences of the United States of America* 105 (42): 16153–58.
